# Lack of kinase-independent activity of PI3Kγ in locus coeruleus induces ADHD symptoms through increased CREB signaling

**DOI:** 10.15252/emmm.201404697

**Published:** 2015-04-16

**Authors:** Ivana D'Andrea, Valentina Fardella, Stefania Fardella, Fabio Pallante, Alessandra Ghigo, Roberta Iacobucci, Angelo Maffei, Emilio Hirsch, Giuseppe Lembo, Daniela Carnevale

**Affiliations:** 1 Department of Angiocardioneurology and Translational Medicine, IRCCS NeuromedPozzilli (IS), Italy; 2 Department of Molecular Biotechnology and Health Sciences, Molecular Biotechnology Center, University of TorinoTorino, Italy; 3 Department of Molecular Medicine, Sapienza University of RomeRome, Italy

**Keywords:** catecholamine, CREB, mouse model, phosphodiesterases (PDEs), stereotactic surgery

## Abstract

Although PI3Kγ has been extensively investigated in inflammatory and cardiovascular diseases, the exploration of its functions in the brain is just at dawning. It is known that PI3Kγ is present in neurons and that the lack of PI3Kγ in mice leads to impaired synaptic plasticity, suggestive of a role in behavioral flexibility. Several neuropsychiatric disorders, such as attention-deficit/hyperactivity disorder (ADHD), involve an impairment of behavioral flexibility. Here, we found a previously unreported expression of PI3Kγ throughout the noradrenergic neurons of the locus coeruleus (LC) in the brainstem, serving as a mechanism that regulates its activity of control on attention, locomotion and sociality. In particular, we show an unprecedented phenotype of PI3Kγ KO mice resembling ADHD symptoms. PI3Kγ KO mice exhibit deficits in the attentive and mnemonic domains, typical hyperactivity, as well as social dysfunctions. Moreover, we demonstrate that the ADHD phenotype depends on a dysregulation of CREB signaling exerted by a kinase-independent PI3Kγ-PDE4D interaction in the noradrenergic neurons of the locus coeruleus, thus uncovering new tools for mechanistic and therapeutic research in ADHD.

See also: **E Darcq & BL Kieffer** (July 2015)

See also: **E Darcq & BL Kieffer** (July 2015)

## Introduction

Phosphoinositide 3-kinases (PI3Ks) are intracellular signaling enzymes activated by various cell-surface receptors, modulating important cellular functions, such as cell survival, proliferation, migration and adhesion (Toker & Cantley, 1997; Bondeva *et al*, 1998). PI3Ks are grouped into three classes on the basis of their structural and biochemical features, and among these, the class I, further divided into IA and IB, has been the most extensively studied. The class IB (PI3Kγ) is composed by the single p110γ catalytic subunit, which is linked to the regulation of G protein-coupled receptor signaling (Stoyanov *et al*, 1995; Patrucco *et al*, 2004) and acting with a dual mechanism involving both kinase-dependent and kinase-independent activities (Carnevale & Lembo, 2012). It has been well demonstrated that PI3Kγ is expressed in the immune and cardiovascular systems, and more recently in the brain (Toker & Cantley, 1997; Oudit *et al*, 2004; Viard *et al*, 2004; Kok *et al*, 2009). However, although PI3Kγ has been extensively investigated in inflammatory and cardiovascular diseases, the exploration of its functions in the brain is just at dawning. On this issue, many years ago, it has been shown that PI3Kγ is present in neurons and that its Akt/PKB signaling is required for the potentiation of L-type channels and for changes in neuronal excitability (Viard *et al*, 2004). More recently, it has been shown that the lack of PI3Kγ in mice leads to an impairment in synaptic plasticity, associated with alterations suggestive of a role in mediating behavioral flexibility (Kim *et al*, 2011).

Several neuropsychiatric disorders, among which schizophrenia, attention-deficit/hyperactivity disorder (ADHD) and behavior addictions are the most common, involve an impairment of behavioral flexibility (Laughlin *et al*, 2011), with related symptoms principally attributed to combined deficits in alerting executive control, involving the prefrontal cortex and the striatum. The resulting poor inhibitory control seems to depend on variations in the monoaminergic system dynamics and particularly on the reciprocal interactions between catecholamines, particularly dopamine (DA) and noradrenaline (NA) (Thapar *et al*, 2005).

Here, we found a previously unreported expression of PI3Kγ throughout the noradrenergic neurons of the locus coeruleus (LC) in the brainstem, serving as a mechanism that regulates its activity of control on attention, locomotion and sociality. Mice with genetic

1 Department of Angiocardioneurology and Translational Medicine, IRCCS Neuromed, Pozzilli (IS), Italy
2 Department of Molecular Biotechnology and Health Sciences, Molecular Biotechnology Center, University of Torino, Torino, Italy
3 Department of Molecular Medicine, Sapienza University of Rome, Rome, Italy
*Corresponding author. Tel: +39 0865 929644; Fax: +39 0865 927575; E-mail: lembo@neuromed.it
**Corresponding author. Tel: +39 0865 929614; Fax: +39 0865 927575; E-mail: daniela.carnevale@neuromed.it
†These authors contributed equally to this work ablation of PI3Kγ exhibit an unprecedented phenotype that mimics the core symptoms of ADHD: They display deficits in attentive domain and are hyperactive, together with an unbalanced catecholaminergic activity in the fronto-striatal areas, receiving projections from the LC. Finally, we propose a novel molecular mechanism that regulates the LC function, mediated by PI3Kγ through a kinase-independent activity and controlling PDE4D to keep homeostatic levels of cAMP. Interestingly, it is conceivable that this mechanism may be relevant for other psychiatric conditions linked to a malfunctioning of the LC, such as chronic stress, opiate addiction and depression.

## Results

### PI3Kγ is expressed in the noradrenergic neurons of the LC

We observed a previously unreported strong expression of PI3Kγ throughout the locus coeruleus (LC) in the brainstem (Fig 1A). In particular, tyrosine hydroxylase (TH)- and dopamine-β-hydroxylase (DBH)-positive cells, characterizing noradrenergic neurons, express PI3Kγ (Fig 1A). The noradrenergic LC mediates a control function on attention, sociality, arousal and activity, which are behavioral states having also a profound impact on cognition, involving the prefrontal cortex (PFC) and the striatum (STR) (Biederman & Spencer, 1999; Berridge & Waterhouse, 2003). Thus, we aimed at assessing the role of PI3Kγ signaling on the behavioral states regulated by the LC and, in order to accomplish this issue, we performed a series of behavioral tests that engage this pathway (Supplementary Table S1).

### PI3Kγ KO mice display poor ability in set-shifting attention

In the first place, we investigated the cognitive responses of PI3Kγ KO mice in the attentional domain, through attentional set-shifting (ASS) test. ASS, adapted to animal models from the original human version, requires attending to relevant stimuli while ignoring irrelevant ones and subsequently shifting the allocation of attention, either within 'dimensions' or between 'dimensions' (Supplementary Tables S2 and S3) (Colacicco *et al*, 2002). Accordingly, mice from all groups required a smaller number of trials to learn simple (SD) and compound (CD) discrimination, compared to other tasks (Fig 1B). Conversely, when both intra- and extra-dimensional shift tasks (respectively, IDS and EDS) were required, PI3Kγ KO mice needed a significantly higher number of trials to reach the criterion, as compared to WT, suggesting poorer ability in set-shifting attention (Fig 1B). This conclusion was also confirmed by the higher number of perseverative errors, considered as a measure of disengaging from an old rule to a new one (Fig 1C). Moreover, PI3Kγ KO required significantly more trials in all the reversal tasks (Fig 1B), thus suggesting an impaired behavioral flexibility.

### PI3Kγ KO mice are hyperactive

Because the LC neurons that project to the PFC may influence spontaneous physical activity, we hypothesized that the presence of PI3Kγ in this brainstem region may also serve as a regulator of this behavioral state. When we tested PI3Kγ KO and WT mice in the open field (OF), PI3Kγ KO displayed significantly greater locomotor activity, manifesting a clear hyperactive phenotype, as shown by distance moved, crossing and vertical activity (Fig 1D–F). The increased activity was also evident in the movement trace patterns: While WT mice moved in a circular fashion following the arena diameter, PI3Kγ KO tended to move with many short stops, frequently changing direction (Fig 1G). No differences emerged in the anxiogenic thigmotaxis behavior (i.e. time spent close to the wall; Supplementary Fig S1).

### Attention deficit and hyperactivity of PI3Kγ KO mice are rescued by MPH

These behavioral phenotypes of PI3Kγ KO mice mimic the core symptoms of ADHD, one of the most prevalent human psychiatric disorders, more recently attributed also to an impairment of LC function (Biederman & Spencer, 1999; Berridge & Waterhouse, 2003). Thus, we hypothesized that methylphenidate (MPH), the drug of choice for the symptomatic treatment of ADHD (Mehta *et al*, 2004) that rescues as well the corresponding behavior in animal models of ADHD (Gray *et al*, 2007; Cao *et al*, 2012) would normalize the phenotype of PI3Kγ KO mice. We found that MPH significantly improved the attention deficit of PI3Kγ KO mice, as evaluated in the ASS test (Fig 1B and C). In particular, PI3Kγ KO mice treated with MPH needed a reduced number of trials to reach the criterion

**Figure 1. PI3Kγ KO mice show attention deficit and hyperactive behavior rescued by MPH.**

A PI3Kγ (green) is constitutively expressed in TH (red)- and DBH (yellow)-positive noradrenergic neurons of the LC. Scale bar, 50 μm. Images are representative of 4 independent experiments.

B Number of trials to reach the criterion in ASS: CD Re $^*P = 0.00057$ (KO veh vs WT veh), $^*P = 0.00012$ (KO veh vs KO MPH), $^*P = 0.0053$ (KO veh vs WT MPH); IDS $^*P = 0.0048$ (KO veh vs WT veh), $^*P = 0.00012$ (KO veh vs KO MPH), $^*P = 0.0011$ (KO veh vs WT MPH); IDS Re $^*P = 0.026$ (KO veh vs WT veh), $^*P = 0.0008$ (KO veh vs KO MPH), $^*P = 0.0043$ (KO veh vs WT MPH); EDS and EDS Re $^*P < 0.000001$ (KO veh vs all other groups).

C Typology of errors during ASS. $^*P < 0.000001$ (KO veh vs all other groups).

D Distance moved in OF. $^*P < 0.000001$ (KO veh vs all other groups).

E Number of crossing in OF: 5-min blocks 1: $^*P = 0.000001$ (KO veh vs WT veh), $^*P = 0.000004$ (KO veh vs KO MPH), $^*P = <0.000001$ (KO veh vs WT MPH); 5-min blocks 2: $^{**}P = 0.000002$ (KO veh vs WT veh), $^*P = 0.00002$ (KO veh vs KO MPH), $^*P = 0.00003$ (KO veh vs WT MPH); 5-min blocks 3: $^*P = < 0.000001$ (KO veh vs all other groups).

F Vertical activity in OF: 5-min blocks 1: $^*P = 0.000002$ (KO veh vs WT veh), $^*P < 0.000001$ (KO veh vs KO MPH), $^*P < 0.000001$ (KO veh vs WT MPH); 5-min blocks 2: $^*P = 0.0034$ (KO veh vs WT veh), $^*P = 0.00013$ (KO veh vs KO MPH), $^*P = 0.00015$ (KO veh vs WT MPH).

G Video tracking showing moving traces of a representative mouse for each group during OF.

Data information: Data were analyzed by ANOVA for repeated measures followed by Bonferroni's *post hoc* test, $n = 8$. Data are means ± SEM.

▶

Figure 1.

(Fig 1B), with less perseverative errors (Fig 1C). WT mice did not show significant alterations after MPH treatment, according to previous studies using similar chronic dosage (Koda *et al*, 2010). Moreover, the MPH treatment suppressed the hyperactivity of PI3Kγ KO, restoring locomotion to levels comparable to that of WT mice (Fig 1D–G).

## Impaired memory in PI3Kγ KO mice

Attention-deficit/hyperactivity disorder patients usually show cognitive deficits in different behavioral patterns. Accordingly, mouse models of ADHD also display impaired memory (Won *et al*, 2011). When we evaluated PI3Kγ KO mice in the Morris water maze (MWM), which tests the spatial memory, we found that, during the acquisition phase, all mice significantly reduced the latency to find the platform (Fig 2A), indicating that PI3Kγ did not affect the normal processes of learning. However, in the probe phase, performed the day after the last acquisition trial, PI3Kγ KO spent significantly less time than WT mice in the quadrant where the platform was located before, indicating an impaired memory of platform location (Fig 2B). With regard to the other parameters considered in this analysis, PI3Kγ KO covered more distance and moved faster, as compared to WT mice (Supplementary Fig S2A and B), further supporting their hyperactive phenotype. During both the acquisition and the probe phases, no difference was found in the anxiogenic thigmotaxis behavior among the experimental groups (Supplementary Fig S2C and D).

## PI3Kγ KO mice show poor social skills with conspecifics

The ADHD psychopathology also unveils poor social adjustment, mainly as a consequence of stigmatization by peers. Thus, we investigated the spontaneous social behavior (SSB) in the home cage, in mice housed each one with a familiar cage mate, during two consecutive days exploiting a procedure known to challenge the social hierarchy (D'Andrea *et al*, 2007). As expected, WT mice modified their behavior according to the change in social hierarchy (Fig 2C and D; Supplementary Fig S3A and B). By contrast, PI3Kγ KO mice spent less time interacting with a conspecific (Fig 2C; Supplementary Fig S3A) and showed an abnormal aggressive grooming (Fig 2D; Supplementary Fig S3B), independently from the environmental challenge. Moreover, also in this test, PI3Kγ KO mice showed a significant increase in the locomotor activity, as compared to WT mice (Supplementary Fig S4A and B). Interestingly, when mice were tested in the elevated plus maze (EPM) test to specifically assess anxiety-like behavior, no difference emerged between two groups (Supplementary Fig S5), suggesting that the higher locomotion observed in PI3Kγ KO mice does not reflex an alteration in the emotional response, but is suggestive of a hyperactive phenotype.

## ADHD-like behaviors of PI3Kγ KO mice depend on a kinase-independent mechanism

During the past years, PI3Kγ has been characterized as an enzyme that can act in a dual manner with kinase-dependent and

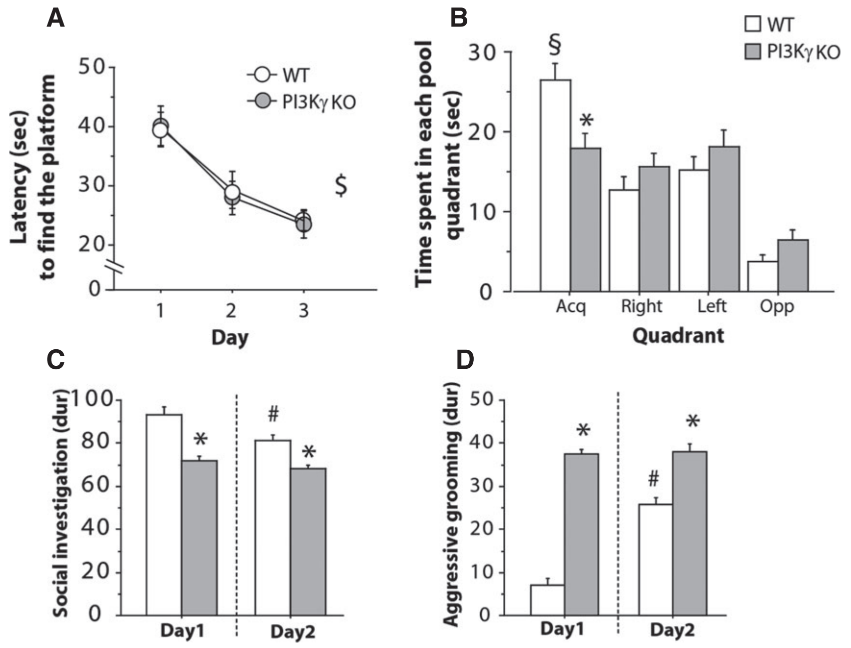

**Figure 2. PI3Kγ KO mice display cognitive impairment and poor social skills.**

A, B Learning curve in MWM (A) ($^\$P < 0.000001$ main effect of time) and probe test for memory retention of MWM test (B) ($^*P = 0.0029$, KO vs WT; $^\S P = 0.000052$ vs all other quadrants). ANOVA for repeated measures followed by Bonferroni's *post hoc* test, $n = 16$. Data are means ± SEM.

C, D Home cage spontaneous social behavior (SSB) showing duration of (C) social investigation, that is, sniffing and grooming the partner in all body regions indicative of affiliative behavior ($^*P = 0.00002$, 0.0011 KO vs WT at day 1 and day 2, respectively; $^\#P = 0.023$ day 2 vs day 1) and (D) aggressive grooming, that is, violent grooming of the animal on the back of the partner ($^*P < 0.000001$, 0.000009 KO vs WT at day 1 and day 2, respectively; $^\#P < 0.000001$ day 2 vs day 1). ANOVA for repeated measures followed by Bonferroni's *post hoc* test, $n = 10$. Data are means ± SEM.

kinase-independent activities (Patrucco *et al*, 2004; Viard *et al*, 2004; Perino *et al*, 2014). The kinase activity of PI3Kγ is mainly linked to the generation of phosphoinositide (3,4,5)-trisphosphate (PIP3), whereas its kinase-independent actions principally affect cAMP levels through the modulation of specific phosphodiesterases (PDEs), the only means of degrading cAMP and maintaining its homeostasis (Patrucco *et al*, 2004; Damilano *et al*, 2011; Perino *et al*, 2011; Ghigo *et al*, 2012). In order to identify the contribution of kinase-dependent and kinase-independent activities of PI3Kγ, we tested the knock-in mouse model expressing a kinase-dead form of PI3Kγ (PI3Kγ KD) (Supplementary Fig S6). Interestingly, PI3Kγ KD exhibited no alterations of the behavioral phenotype both in the attention domain (Supplementary Fig S6A and B) and in the locomotor activity (Supplementary Fig S6C–E), which were clearly overlapping to that of WT mice, suggesting that the ADHD-like behaviors showed by PI3Kγ KO mice should be mediated by a kinase-independent mechanism.

### PI3Kγ in the LC control PDE4D activity

Interestingly, cAMP levels are the main regulator of the LC function (Nestler & Aghajanian, 1997; Han *et al*, 2006). Since the lack of PI3Kγ, but not of its kinase activity, affects the brain domains organized and regulated by the noradrenergic LC, we reasoned that a mechanism affecting cAMP metabolism in the LC could be engaged in our model. Several isoforms of PDEs are known, but PDE4 is the cAMP-specific PDE that is prevalent in the brain (Kleppisch, 2009). Interestingly, PI3Kγ is also a critical activator of membrane-bound PDE4 in the myocardium (Ghigo *et al*, 2012). Thus, we examined whether PI3Kγ controls PDE4 in the LC. The major PDE4 isoenzymes of the mouse brain were immunoprecipitated from membrane and cytosolic fractions of PI3Kγ KO, KD and WT LC punches, and IP pellets were assayed for cAMP PDE activity (Fig 3A). The catalytic activity of particulate and soluble PDE4D was significantly lower in PI3Kγ KO than in WT tissues, whereas no difference was found in PI3Kγ KD as compared to WT (Fig 3A). PDE4A and PDE4B activities were unchanged in PI3Kγ KO (Fig 3A). Thus, the presence of PI3Kγ is required to selectively control PDE4D activity in both membrane and cytosolic compartments of the LC (Fig 3A). The finding that PDE4D physically interacts with PI3Kγ when we immunoprecipitated this latter from LC punches (Fig 3B) further strengthens the evidence that suggest that PI3Kγ signaling in the LC controls cAMP levels and homeostasis through PDE4D.

### Lack of PI3Kγ turns into an increased CREB signaling in the LC and catecholaminergic activity in projection areas

The LC neuronal excitability is regulated by the activation status of the transcription factor cAMP response element binding protein (CREB) and its binding to the promoter of target genes (Nestler & Aghajanian, 1997; Han *et al*, 2006; Cao *et al*, 2010). Thus, we conceived that the reduced PDE4D activity observed in PI3Kγ KO could turn into dysregulated cAMP and increased CREB activation. Indeed, we found and increased pCREB/CREB in the LC of PI3Kγ KO (Fig 3C–E), suggestive of an increased activation of the noradrenergic activity of the LC, possibly determining an inhibitory effect on the dopaminergic transmission in the projecting brain areas. Accordingly, we measured NA and DA in PFC and STR, finding a significant increase in NA, with a concomitant reduction in DA (Fig 3F). Conversely, both NA and DA remained unchanged in the LC of PI3Kγ KO mice (Fig 3F). In order to exclude the possibility that the NA/DA balance in projecting areas could be due to other areas controlling the monoaminergic system, we evaluated the presence of PI3Kγ in the substantia nigra (SN) and in the ventral tegmental area (VTA), the major sources of dopaminergic neurons. As shown in Supplementary Fig S7A, we did not find expression of PI3Kγ in dopaminergic neurons of both SN and VTA (Supplementary Fig S7A).

### Selective inhibition of CREB in the LC rescues the ADHD-like behavior of PI3Kγ KO mice

It has been reported that *in vivo* blockade of CREB activity in the LC prevents its neuronal hyperexcitability (Han *et al*, 2006). In order to better understand the role of the PI3Kγ-mediated regulation of cAMP–CREB signaling in the LC, we selectively injected the LC of PI3Kγ KO mice with an adeno-associated viral vector carrying dnCREB or GFP alone as a control, by bilateral stereotactic surgery (Fig 4A; Supplementary Fig S8). Three weeks later, we obtained an effective expression of the viral vector (Fig 4A). We found that, by antagonizing CREB in the LC, we were able to rescue the attention deficit (Fig 4B and C) and the hyperactivity (Fig 4D–F), displayed by PI3Kγ KO mice, while ameliorating their poor social skills (Fig 4G and H; Supplementary Fig S9). Moreover, we found a restoration of the NA/DA unbalance in the PFC and STR in PI3Kγ KO mice injected with AAVdnCREB, as compared to PI3Kγ KO AAVGFP control mice (Supplementary Fig S10).

**Figure 3. Lack of PI3Kγ in the LC dysregulates PDE4D independently from kinase activity and consequent cAMP–CREB activation.**

A Kinase-independent activity of PI3Kγ selectively regulates PDE4D activity in both membrane (mem) and cytosolic (cyt) compartments of LC ($*P = 0.015$ and $*P = 0.046$ in mem and cyt vs WT and $^{\#}P = 0.0037$ and $^{\#}P = 0.030$ in mem and cyt vs KO; ANOVA followed by Bonferroni's *post hoc* test, $n = 5$). Data are means ± SEM.

B PI3Kγ is constitutively associated with PDE4D, as shown by the co-IP of the two proteins in homogenates from bilateral LC punches ($n = 5$ for each experimental group).

C, D CREB (C, red) expression and pCREB (D, red) expression in TH (yellow)-positive noradrenergic neurons of the LC in PI3Kγ KO and WT mice ($n = 5$ for each experimental group). Scale bar, 50 μm.

E WB analysis of nuclear/cytosol extract from LC punches of PI3Kγ KO and WT mice, showing increased levels of pCREB in the nucleus of KO LC ($n = 5$ for each experimental group).

F Levels of NA and DA in LC and PFC/STR of PI3Kγ KO and WT mice (unpaired two-tailed Student's *t*-test, $n = 8$, $*P = 0.033$ NA in PFC and STR; $*P = 0.029$ DA in PFC and STR). Data are means ± SEM.

Source data are available online for this figure.

A
WT PI3Kγ KO PI3Kγ KD
PDE Activity (cpm/IP)
PDE Activity (cpm/IP)
IP PDE4A (mem)
IP PDE4A (cyt)
IP PDE4B (mem)
IP PDE4B (cyt)
PDE Activity (cpm/IP)
IP PDE4D (mem)
IP PDE4D (cyt)
B
IP PI3Kγ
75kDa
WT PI3Kγ KO
PDE4D
WB
C
CREB TH DAPI
WT
PI3Kγ KO
D
WT
PI3Kγ KO
pCREB TH DAPI
E
nuclear
cytosol
WT PI3Kγ KO WT PI3Kγ KO
pCREB
43kDa
F
LC
PFC and STR
WT
PI3Kγ KO
Pmol NA/mg tissue
Pmol DA/mg tissue

**Figure 3.**

### Constitutive activation of CREB in the LC of WT mice resembles ADHD-like phenotype

In order to assess further the relevance of the cAMP–CREB signaling in the behavioral alterations related to ADHD, we overexpressed a constitutively active CREB in the LC of WT mice, by an adeno-associated viral vector carrying caCREB (or GFP as a control), as described above. Interestingly, WT mice with AAVcaCREB induced behavioral alterations overlapping those observed in PI3Kγ KO mice. In particular, they needed more trials to reach the criterion (Fig 5A) and made more perseverative errors (Fig 5B) in the ASS compared to AAVGFP control mice. Moreover, AAVcaCREB mice were hyperactive (Fig 5C–E) and displayed poor social skills (Fig 5F and G; Supplementary Fig S11). Further supporting the ADHD-like phenotype, we found that the injection of AAVcaCREB in WT mice induced an alteration of NA and DA in the PFC and STR (Supplementary Fig S12) similar to that observed in PI3Kγ KO mice. Overall, these results clearly associate the ADHD phenotype, previously characterized in PI3Kγ KO mice, to a dysfunction of the cAMP–CREB pathway in LC.

## Discussion

In the present work, we have identified that PI3Kγ in the brainstem is expressed in the LC and that its absence reproduces typical ADHD-like behaviors. In particular, PI3Kγ KO mice exhibit deficits in sustained attentional shifting capacity and are hyperactive. In addition, PI3Kγ KO mice show impaired spatial memory and social dysfunctions characterized by poor skills and competences with conspecifics. This behavioral phenotype is accompanied with neurochemical alterations, reflected by an unbalanced catecholaminergic activity in the PFC and STR, the brain areas receiving projections from the LC.

The diagnostic criteria in ADHD are currently based on behavioral symptoms rather than on molecular or neuroanatomical indicators. Thus, the development of mouse models recapitulating all the behavioral features of such disease is crucial to study the molecular bases underlying ADHD etiology. However, since ADHD is a heterogeneous disorder, suggested to result from combinations of genetic and environmental factors, we should be careful when translating behavioral data from animal models to humans. Murine models can mimic only certain aspect of the complex symptomatology of ADHD, but may still provide feasible hypotheses regarding the underlying causes of specific ADHD symptoms. Novel insights into disease mechanisms could be the only means for the design of targeted therapeutic treatments that indeed, so far, are mainly based on drugs that mildly relieve from some of the symptoms.

Catecholamines have an essential role in the emergence of ADHD. Despite several works focused on DA, it is known that also NE strongly affects cognitive abilities via postsynaptic α2A-adrenoreceptors (α2A-ARs) (Arnsten & Goldman-Rakic, 1985). Indeed, NA activation is known to profoundly affect the performance of attention, especially the maintenance of arousal, a cognitive function known to be poor in ADHD (Biederman & Spencer, 1999). In the present study, the molecular analysis of PI3Kγ KO mice revealed a main mechanism, involving LC-NA system leading to an increased NA transmission in fronto-striatal structures, predominantly regulated by NA neurons, able to exert an inhibitory control over DA transmission. This neurobiological perspective fits very well with the current neuropsychological, genetic, imaging and pharmacological data emerging in ADHD research, providing compelling support for a noradrenergic hypothesis of ADHD and demonstrating that very high levels of catecholamine release disrupt cognitive functions of the PFC (Arnsten & Goldman-Rakic, 1985; Franowicz *et al*, 2002). Our findings show that this mechanism is mediated by the constitutive presence of PI3Kγ in the LC where, through a kinase-independent mechanism, it controls PDE4D activity to keep homeostatic levels of cAMP. A balanced cAMP–CREB signaling pathway is well known to be needed for a proper working of the LC.

The ultimate goal of understanding the pathophysiology of the disorder is to develop therapeutic interventions, which would improve or restore physiological brain activity and, ultimately, lead to a rescue of the behavioral phenotype. Here, we have showed that a tonic upregulation of cAMP–CREB activity in the LC of PI3Kγ KO mice turns into a behavioral phenotype having face validity with ADHD symptoms. Interestingly, a selective genetic intervention obtained by a stereotaxis-driven inoculation of AAV carrying dnCREB in the LC rescued such behavioral alterations in PI3Kγ KO mice. Finally, the same stereotaxis manipulation, applied for inoculation of AAV overexpressing CREB in WT LC, reproduces the ADHD-like phenotype observed in PI3Kγ KO mice. These findings sustain the hypothesis whereby PI3Kγ controls NA transmission of the LC, by regulating the homeostasis of cAMP levels and the

**Figure 4. Selective inhibition of CREB in the LC rescues the ADHD-like behaviors of PI3Kγ KO mice.**

A Target sites of viral injection are shown in black in the section of brain atlas map comprising LC. GFP expression in the injected site of a representative animal shown at two different magnification scales.

B Number of trials to reach the criterion in ASS: CD Re $^*P = 0.0028$ and 0.0021 vs WT AAVGFP and KO AAVdnCREB; EDS Re $^*P = 0.000003$ and 0.000009 vs WT AAVGFP and KO AAVdnCREB; in the all other tasks: $^*P < 0.000001$ (KO AAVGFP vs other two groups).

C Typology of errors in ASS test: $^*P = < 0.000001$ vs other two groups.

D Distance moved in OF: 5-min blocks 1 and 2: $^*P < 0.000001$ vs other two groups; 5-min blocks 3: $^*P = 0.0032$, 0.000029 (KO AAVGFP vs WT AAVGFP and KO AAVdnCREB, respectively).

E Number of crossing in OF: $^*P < 0.00001$ KO AAVGFP vs other groups.

F Vertical activity in OF: 5-min blocks 1: $^*P = 0.000009$ (KO AAVGFP vs WT AAVGFP), 0.000057 (KO AAVGFP vs KO AAVdnCREB); 5-min blocks 2: $^*P = 0.0014$ (KO AAVGFP vs WT AAVGFP) and 0.00035 (KO AAVGFP vs KO AAVdnCREB).

G, H SSB showing duration of (G) social investigation (day 1: $^*P = 0.000044$ and 0.00010, KO AAVGFP vs WT AAVGFP and KO AAVdnCREB, respectively) and (H) aggressive grooming (day 1: $^*P = 0.00020$ and 0.011, KO AAVGFP vs WT AAVGFP and KO AAVdnCREB, respectively; day 2: $^*P = 0.00019$ and 0.00068, KO AAVGFP vs WT AAVGFP and KO AAVdnCREB, respectively; $^{\#}P = 0.0015$ day 2 vs day 1).

Data information: ANOVA for repeated measures followed by Bonferroni's *post hoc* test, $n = 10$. Data are means ± SEM.

consequent activity of CREB transcription factor, the main regulator of LC neuronal excitability.

Overall, our results propose a novel molecular mechanism that regulates the LC function and is mediated by the constitutive presence of PI3Kγ through a kinase-independent activity that controls PDE4D to keep homeostatic levels of cAMP. Besides the relevance of this pathway in ADHD and the perspective to use PI3Kγ KO mice as a model of ADHD, our findings put perspective into a novel molecular pathway that balances the homeostasis of cAMP in the LC, allowing its relay activity, known to be important also in chronic perturbations, such as chronic stress, opiate addiction and depression (Nestler & Aghajanian, 1997; Mazei-Robison & Nestler, 2012).

Further supporting an essential role of PI3Kγ in the phenotypes related to behavioral flexibility, it has also been reported a genetic

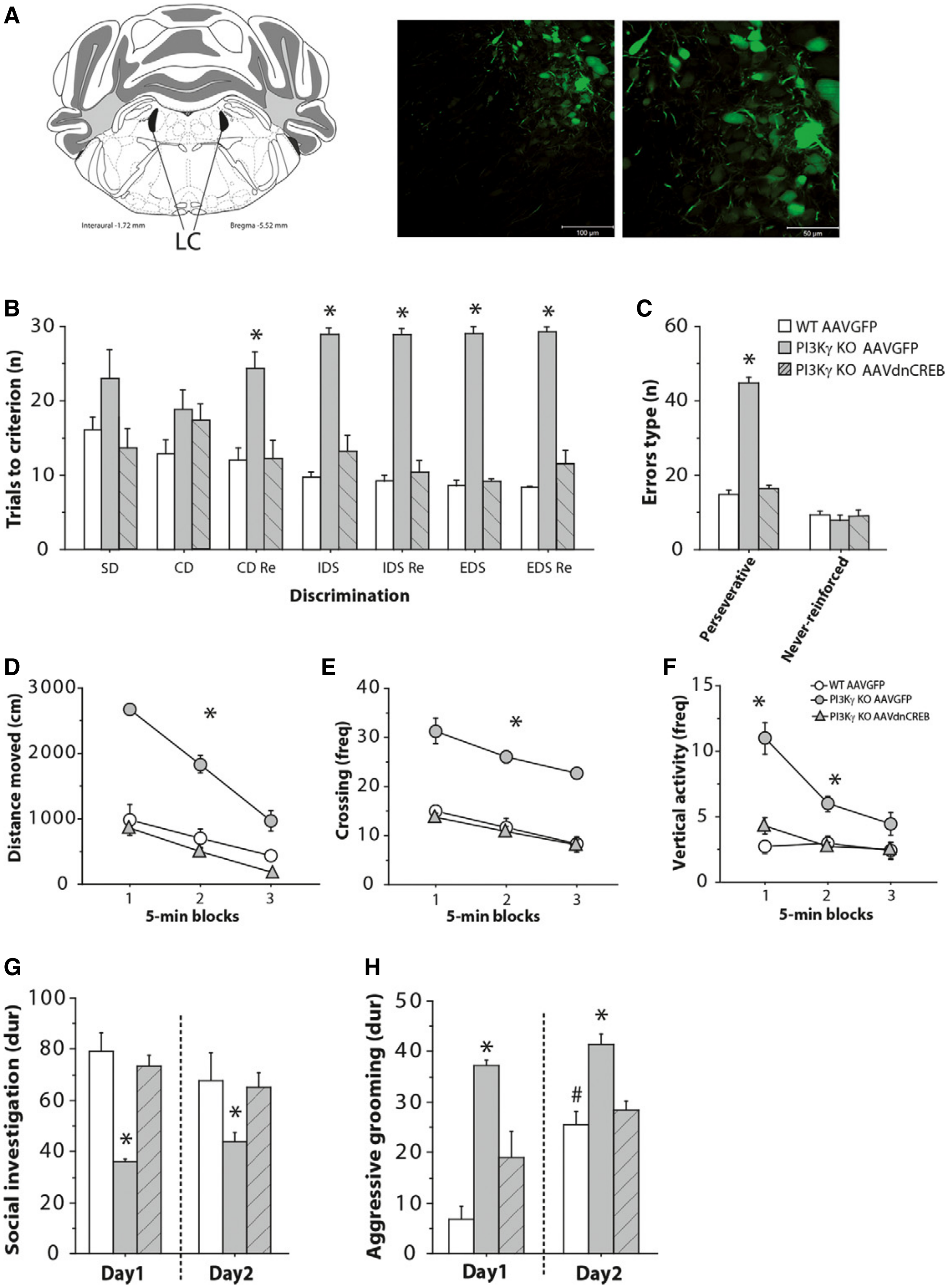

Figure 4.

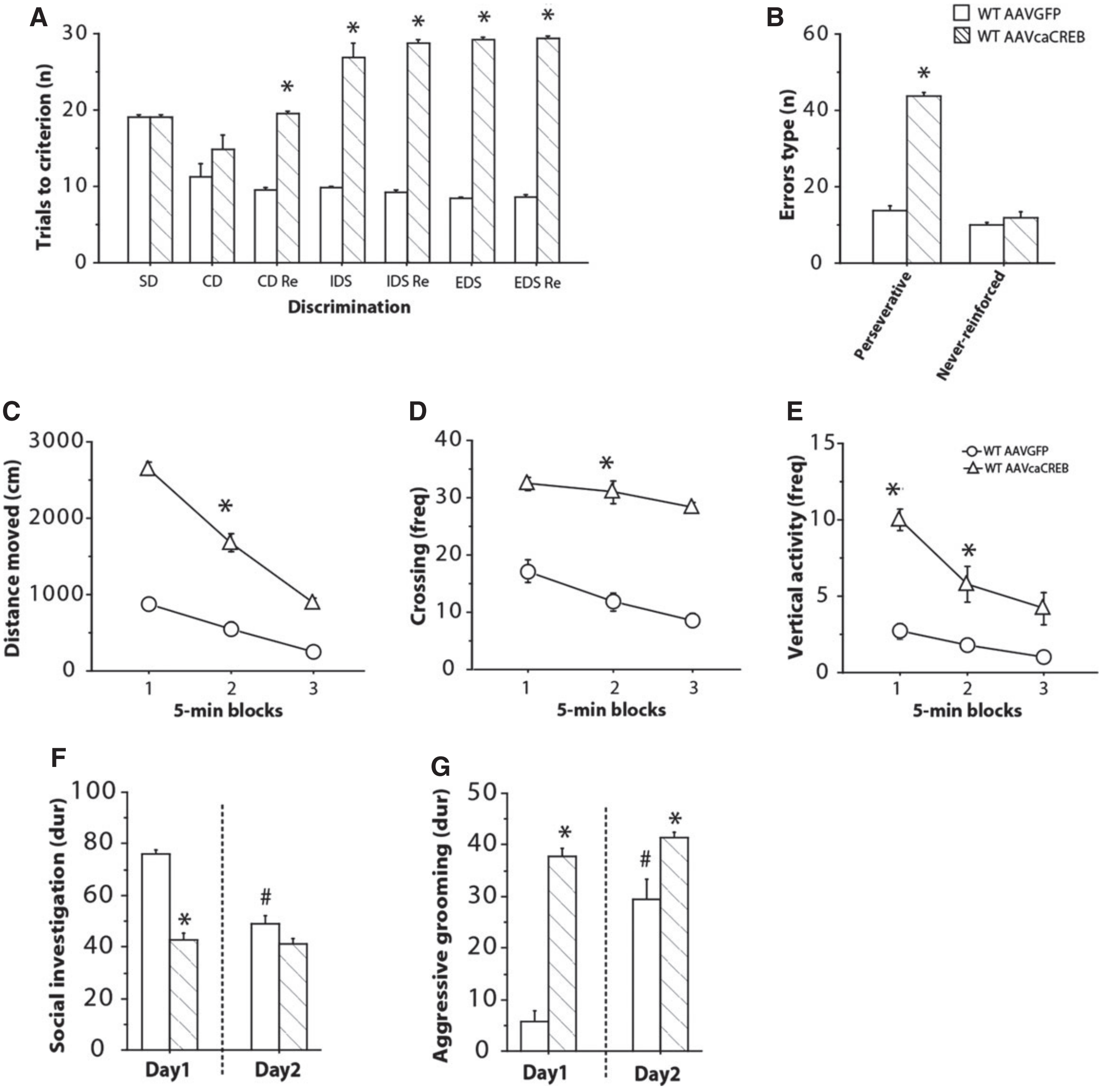

**Figure 5. Selective overexpression of a constitutively active CREB in the LC induces ADHD-like behaviors in WT mice.**

A Number of trials to reach the criterion in ASS: CD Re, IDS, IDS Re, EDS, EDS Re: $^*P < 0.000001$, WT AAVcaCREB vs WT AAV-GFP.

B Typology of errors in ASS test: $^*P < 0.000001$, WT AAVcaCREB vs WT AAV-GFP.

C Distance moved in OF: 5-min blocks 1 and 2: $^*P < 0.000001$; 5-min blocks 3: $^*P = 0.000078$, WT AAVcaCREB vs WT AAV-GFP.

D Number of crossing in OF: 5-min blocks 1: $^*P = 0.000001$; 5-min blocks 2: $^*P = 0.000001$; 5-min blocks 3: $^*P = 0.000001$, WT AAVcaCREB vs WT AAVGFP.

E Vertical activity in OF: 5-min blocks 1: $^*P = 0.000008$; 5-min blocks 2: $^*P = 0.016$, WT AAVcaCREB vs WT AAVGFP.

F, G SSB showing duration of (F) social investigation ($^*P = 0.000001$, WT AAVcaCREB vs WT AAVGFP; $^{\#}P = 0.000007$ day 2 vs day 1) and duration of (G) aggressive grooming ($^*P < 0.000001$ and $^*P = 0.017$, WT AAVcaCREB vs WT AAVGFP on day 1 and day 2 respectively; $^{\#}P = 0.000022$ day 2 vs day 1).

Data information: Data were analyzed by ANOVA for repeated measures followed by Bonferroni's *post hoc* test, $n = 5$. Data are means ± SEM.

link between PI3Kγ dysfunction and mental disorders, particularly autism. Indeed, the gene encoding PI3Kγ, *PI3KCG*, is located within the autism susceptibility locus *AUTS1* on chromosome 7q22 (Kratz *et al*, 2002; Serajee *et al*, 2003). On this issue, it is important to notice that, for long time, autism and, more in general, autism spectrum disorders (ASD) have been considered somewhat completely distinct from ADHD. However, this belief has been reevaluated when many observational data brought to light a frequent co-occurrence of the two conditions, opening the possibility that some symptoms found in ASD meet also the criteria of ADHD. With our findings that strongly suggest PI3Kγ as a crucial player of ADHD, we propose that the relationship between this psychiatric syndrome and ASD needs a more profound molecular and mechanistic investigation in mouse models, in order to open novel therapeutic perspectives.

## Materials and Methods

### Mice

PI3Kγ-deficient (PI3Kγ KO) mice used in all experiments were either in a 129Sv/Pas or in a C57Bl/6J background; knock-in with catalytically inactive PI3Kγ (PI3Kγ KD) mice was on C57Bl/6J background. PI3Kγ KO, PI3Kγ KD and their respectively wild-type (WT) control mice were generated as previously described (Patrucco *et al*, 2004; Kok *et al*, 2009; Perino *et al*, 2011; Carnevale & Lembo, 2012; Ghigo *et al*, 2012). The main phenotype of PI3Kγ KO mice, that is, ADHD, was present and comparable in both strains. Accordingly, we used PI3Kγ KO mice in the C57Bl/6J background as matched experimental group of PI3Kγ KD, whereas we carried out all the other experiments on 129Sv/Pas background.

The animals, aged 6–8 weeks, were housed in an air-conditioned room (temperature 21 ± 1°C, relative humidity 60 ± 10%) with lights on from 08.00 to 20.00 h, in same-sex groups of 4 individuals in 42 × 17 × 14 cm Plexiglas boxes with a metal top and sawdust as bedding, and with pellet food (Enriched standard diet, Riper, Vandoies, BZ, Italy) and tap water *ad libitum*. Regarding the number of mice used for each experimental setting, we referred to sample sizes commonly used for the same kind of studies. All animal handling and experimental procedures were performed according to the European Community guidelines (EC Council Directive 2010/63) and the Italian legislation on animal experimentation (Decreto L.vo 116/92). The protocol was approved by the Italian Ministry of Health (Permit number 58/2012-B). All efforts were made in order to minimize suffering. In particular, the principles of Replacement, Reduction and Refinement (i.e., the 'three Rs') have been applied to all experiments.

### Behavioral tests

Ten mice per genotype were evaluated for each behavioral test. All tests were carried out between 09.30 and 16.30 h, by blinded investigators. Animals were transferred to the experimental room at least 45 min before the tests in order to let them acclimatize to the test environment. After testing each animal, each apparatus was thoroughly cleaned with cotton pads wetted with 30% ethanol. Behavior was videorecorded using a digital video camera (ICD-49E, Ikegami, Japan). The behavioral scoring was carried out using a computer-based video-tracking system Ethovision XT (Noldus, Wageningen, the Netherlands) and the commercial software Observer XT (Noldus). All scores were assigned from the same observer who was unaware of animal genotype and/or treatment.

#### *Attentional set-shifting*

We adopted the attentional set-shifting task, developed by Colacicco *et al* (2002). Apparatus and general procedure: The apparatus was an opaque PVC U-shaped box with a grid floor and a transparent Plexiglas lid (45 cm × 30 cm × 15 cm). Two identical choice compartments (15 cm × 15 cm) at one end of the apparatus could be accessed through sliding doors from a starting compartment (30 cm × 30 cm). A cylindrical food cup (40 mm diameter, 35 mm high) in each choice compartment could be baited with a small piece of cereal (30 mg; Honey Nut Loop, Kellogg's). The food was then covered with a layer of medium-scented digging (20 mm). The presence or absence of food reward in a cup was indicated by either tactile (type of digging medium) or olfactory stimuli (scent of the digging medium). The entire procedure took 5 days, a 1-day habituation period and a 4-day testing period. Habituation: On the day before testing, mice were given access to the apparatus for 30 min. Following this preliminary exposure, mice were trained to dig into food-baited bowls during a series of 9 consecutive trials. Testing: In order to avoid potential biases, the odor presentation was randomized across cage mates. A stage was considered complete when the mouse achieved a criterion of 8 correct trials out of 10. Trials were concluded after 5 min if no choice was made, and marked as incomplete. A session would continue until the animal ceased responding. However, one hour of responding resulted in an average of approximately 30 trials per day per subject. Briefly, a trial was initiated by raising the sliding wall to give the mouse access to the two digging bowls, only one of which was baited. Mice were required to dig into a rewarded bowl in order to obtain highly palatable food pellets. Digging bowls varied across two dimensions (digging medium and scent). During simple discrimination (SD), mice had to learn to discriminate between two different odors or digging media. After this stage, mice were required to perform a compound discrimination (CD), during which the baited stimulus of the previous stage was presented together with another, newly introduced, irrelevant stimulus of the other dimension. Despite the presence of the new stimulus, the correct and incorrect exemplars remained constant. At the end of this stage, mice had to perform CD reversal (CDR) learning. For the reversal, the exemplars and the relevant dimensions were unchanged: The mouse had to learn that the previously correct stimulus was now incorrect. For both intra-dimensional shift (IDS) and extra-dimensional shift (EDS), new exemplars of both dimensions were used. In the IDS, the relevant dimension is the same as before, whereas in the EDS, the mouse had to shift attention to the previous irrelevant dimension. The order of discriminations was always the same, but dimensions the pairs of exemplars were equally represented within groups and counterbalanced between groups as far as possible. Errors during testing were broken down into perseverative errors and never-reinforced errors. Perseverative errors were scored when a mouse continued to use the previously relevant but currently irrelevant rule. In 5 out of every 10 consecutive trials, the mouse was allowed to respond this way. Never-reinforced errors were scored when mice made incorrect choice using a rule that has never been rewarded previously.

#### *Open field*

Mice were individually placed in the center of a cubic arena (40 × 40 × 40 cm) made of black Plexiglas and allowed to freely explore for 15 min. The open field box was ideally divided into 25 squares and ideally partitioned into a central portion (26.4 × 26.4 cm) and a peripheral one (i.e., thigmotaxis—time spent near the walls), identified as the remaining parts of the arena. When data were analyzed, each session was subdivided in 3 5-min blocks and time spent in each portion of the arena, total distance moved as well as latency, frequency and duration of locomotion (crossings of squares, vertical activity, i.e., rearing and wall rearing), and self-directed behaviors (self-grooming) were scored.

#### *Morris water maze*

The water maze apparatus consisted of a white Plexiglas circular pool 88 cm in diameter and 33 cm in height, filled with water and kept at a temperature of 26 ± 1°C. A plastic transparent platform (8 cm in diameter) was placed 0.5 cm below the water surface and 10 cm from the edge of the pool. The ability of experimental subjects to identify and reach a visible platform was tested in one visual cued version of the task which preceded the 3 days of acquisition phase. Each day, mice underwent 3 trials during which they were allowed to freely swim for 60 s or until they found and climbed onto the platform; each trial was spaced from the other by a 40-min inter-trial interval. In the visual phase of the task, a visual cue was always located on the platform and the platform position was changed in each trial, whereas during the acquisition phase, the platform position remained fixed for all the trials in a 'target quadrant'. Platform finding was defined as staying for at least 3 s on it. During the acquisition phase, mice that did not find the platform were trained in locating it by placing them on the platform for 10 s at the end of the trial. On the last day (probe phase), each mouse was tested for memory retention in a 60-s probe trial during which the platform was removed from the pool and the time spent in the area of the maze where the platform was located was scored. For the acquisition phases, the variables recorded were latency to reach the platform, mean swimming speed, distance moved and thigmotaxis. For the probe phases, the variables recorded were time spent in each quadrant, mean swimming speed, distance moved and thigmotaxis.

*Spontaneous social behavior*

The spontaneous social behavior was recorded in the home cage according to the paradigm previously described (D'Andrea *et al*, 2007). Ten WT and 10 KO cages (two mice in each cage) were observed for a single 30-min session on two consecutive days. Since data collected concerning 20 mice per experimental group have been found to be dependent (significant effect of random cage factor in the mixed model ANOVA), only one mouse, randomly selected from each cage, has been considered in the analysis ($n = 10$). Seven days before the first day of observation, the cages were cleaned and one of the two experimental subjects was marked with a blue, scentless and nontoxic felt pen in order to discriminate the two animals during data collection. During the first day of observation, no manipulation was performed. On the second day, the cage sawdust was changed with clean sawdust just before the session of observation. This procedure is known to elicit aggressive behavior and to challenge social hierarchy. Social behavior was scored during a single 30-min session each day. The behavioral categories and elements scored for both frequency and duration were social investigation (sniffing and grooming the partner in all body regions indicative of affiliative behavior) and aggressive grooming (violent grooming of the animal on the back of the partner).

*Elevated plus maze*

The elevated plus maze comprised two open arms (30 cm × 5 cm × 0.25 cm) and two closed arms (30 cm × 5 cm × 15 cm) extended from a common central platform (5 cm × 5 cm). The apparatus was constructed from Plexiglas (black floor, transparent walls) and elevated to a height of 60 cm above the floor level on a central pedestal. Mice were individually placed on the center of the platform facing an open arm and allowed to explore freely the maze for 5 min. Behavioral parameters scored were time spent in open and closed arms and percentage of open time.

**Drug administration**

Mice were randomized to receive methylphenidate (3 mg/kg) or vehicle, daily administrated by gavage for 21 consecutive days.

**PDE4 determination**

*Protein extraction*

Total membranes and cytosolic fractions were prepared by homogenization of liquid nitrogen-frozen LC in 120 mmol/l NaCl, 50 mmol/l Tris–HCl (pH 8.0), protease inhibitor complete (Roche Applied Science, Indianapolis, IN) and phosphatase inhibitors (50 mmol/l sodium fluoride, 1 mmol/l sodium orthovanadate and 10 mmol/l sodium pyrophosphate). Lysates were incubated on ice for 30 min and then centrifuged at 1,500 *g* for 5 min at 4°C. Membrane and cytosolic fractions were then separated by centrifugation of supernatants at 38,000 rpm for 1 h at 4°C in a SW55Ti rotor (Beckman Coulter). Supernatants (cytosolic fraction) were collected, and pellets (membrane fraction) were solubilized in ice-cold lysis buffer, supplemented with 1% Triton X-100. Protein concentration was determined by the Bradford method, and extracts were used for immunoprecipitation and then assayed for PDE activity.

*Immunoprecipitation*

For immunoprecipitation assays, 150 μg of pre-cleared extracts was incubated with 20 μl of a 1:1 slurry of protein A- or G-Sepharose (Amersham Biosciences, Buckinghamshire, UK) and 1 μg of antibody/mg of protein for 2 h at 4°C. Immunocomplexes were then extensively washed with lysis buffer for PDE activity assay.

*PDE assay*

Phosphodiesterase activity in immunoprecipitates was measured according to the two-step method of Thompson and Appleman. In brief, immunoprecipitations were assayed in a total volume of 200 μl of reaction mixture containing 40 mmol/l Tris–HCl (pH 8.0), 1 mmol/l $MgCl_2$, 1.4 mmol/l 2-mercapto-ethanol, 1 μmol/l cAMP (Sigma-Aldrich, Saint Louis, MO) and 0.1 μCi of [3H]cAMP (Amersham Bioscience, Buckinghamshire, UK) for 45 min at 33°C. In order to stop the reaction, samples were boiled at 95°C for 3 min. The PDE reaction product 5′-AMP was then hydrolyzed by incubation of the assay mixture with 50 μg of Crotalus atrox snake venom for 15 min at 37°C (Sigma-Aldrich, Saint Louis, MO). The resulting adenosine was separated by anion exchange chromatography using 400 μl of a 30% (w/v) suspension of Dowex AG1-X8 resin (Bio-Rad, Segrate, Milano, Italy). The amount of radiolabelled adenosine in the supernatant was quantified by scintillation counting (Ultima Gold scintillation liquid from Perkin Elmer, Waltham, MA).

*Antibodies*

Anti-PDE4A, anti-PDE4B and anti-PDE4D antibodies used for immunoprecipitation were from Abcam (Cambridge, UK).

**Immunofluorescence**

Mice were anesthetized with farmotal and transcardially perfused with saline containing 4% paraformaldehyde. Brains were removed and immersed in fixative for six hours at 4°C and then cryoprotected in 20% sucrose overnight at 4°C. Thirty-micrometer-thick floating frozen section were cut using a cryostat (Leica) and then sequentially incubated with primary antibody: rabbit anti-Creb 1:1,600 (Cell Signaling), rabbit anti p-Creb 1:500 (Cell Signaling), mouse anti-PI3Kγ produced as previously described (Damilano *et al*, 2011; Perino *et al*,

2011) 1:100 in MOM kit (Vector), goat anti-dopamine beta hydroxylase 1:800 (Abcam) and sheep anti-tyrosine hydroxylase 1:800 (Millipore). The secondary antibodies used were Alexa 488 1:200, Cy3 1:200 and Alexa 647 1:100 from Jackson Immunoresearch. Slides were cover-slipped with DAPI-containing medium (Vector).

### Confocal microscopy analysis

All cover-slipped, mounted tissue sections were scanned using a Zeiss 780 confocal laser scanning microscope with a Zeiss ECPLAN-NEOFLUAR 20×/0.50 M27, ECPLAN-NEOFLUAR 5×/0.16 or ECPLAN-NEOFLUAR 40×/1.30 M27 oil immersion objective (Carl Zeiss Microimaging Inc.). We used a 405-nm diode laser to excite DAPI, a 488-nm argon laser to excite Alexa Fluor 488, a 543-nm HeNe laser to excite Cy3 and a 633-nm HeNe laser to excite Alexa Fluor-647. Z-stack projections and pseudo-coloring were performed using ZEN software (Carl Zeiss Microimaging, Inc.).

### Western blot and immunoprecipitation

Mouse brains were rapidly removed and chilled in ice-cold buffer containing (in mM) 126 NaCl, 5 KCl, 1.25 $NaH_2PO_4$, 2 $CaCl_2$, 2 $MgCl_2$ and 10 D-glucose, pH 7.4. The LC was obtained from coronal brain slide of ∼1 mm, cut with McIlwain tissue chopper. Bilateral punches of the LC from two slices of the same animals were homogenized by dunce homogenizer with NE-PER nuclear and cytoplasmatic extraction kit (Thermo Scientific). Protein content was determined by the BCA method. Fifty micrograms of proteins from each sample was separated by SDS–polyacrylamide gel electrophoresis using a Tris/Glycine/SDS buffer (Bio-Rad) and transferred to a PVDF membrane (Bio-Rad). Membranes were blocked using BSA (Sigma) and then incubated overnight at 4°C with primary antibody pCreb (ser133; Cell Signaling) 1:400 and secondary antibody goat anti-rabbit (Santa Cruz).

For immunoprecipitation, 250 μg of total proteins extracted from bilateral LC punches was incubated with 20 μl protein A/G agarose plus (Santa Cruz), 1 μg of anti-PI3kg antibody/mg of protein overnight at 4°C. Immunocomplexes were washed with lysis buffer and resolved by SDS–PAGE followed by Western blotting using the antibody PDE4D (Acris) 1:400.

Secondary antibodies were from Santa Cruz, and signal detection was performed with ECL Kit (Amersham) and analyzed with Chemidoc XRS Imaging System (Bio-Rad).

### Noradrenaline and dopamine determination

Locus coeruleus was homogenized by sonication in lysis buffer 1 mM EDTA and 0.1 N HCl. Dopamine and Noradrenaline levels were measured with a high-sensitivity ELISA kit (IBL International), following the manufacturer's instructions.

### Surgical procedure

Mice were anesthetized with an intraperitoneal injection of ketamine (90 mg/kg) and xylazine (10 mg/kg), and thermoregulation was provided through a thermostat-regulated heating pad (Harvard apparatus) and monitored through a rectal thermometer. The head was shaved and cleaned with iodine before incision. After making a skin incision of about 1 cm, mice were placed on the motorized stereotaxic apparatus (Stoelting). The point 0 (bregma) and subsequently the respective coordinates for injection into the LC (*x*-5.52 *y*-0.85 *z*-3.50) were established as described by Franklin and Paxinos (Franklin & Paxinos, 1996). Mice were randomized in experimental groups and injected with the following viral vectors: adeno-associated virus (AAV)-green fluorescent protein (GFP) as a control, AAV-dnCREB (a dominant-negative mutant of CREB) or AAV-caCREB (constitutively active mutant of CREB) as specified in each figure. Subsequently, skull and skin were closed with dental cement and a 6.0 (Ethicon) suture. After surgery, mice were monitored in an incubator at a controlled temperature and were treated with antibiotic for any post-operative infection.

#### The paper explained

**Problem**
PI3Ks are enzymes characterized by both lipid and protein kinase activity, which once activated modulate important cellular functions, affecting cell survival, proliferation, migration and adhesion. Among these, PI3Kγ, which is mainly linked to regulation of G protein-coupled receptor signaling, acts with a dual mechanism involving both kinase-dependent and kinase-independent activity, the first being mainly linked to regulation of intracellular levels of phosphoinositide (3,4,5)-trisphosphate (PIP3) and the second affecting cAMP levels through modulation of specific PDE isoforms activity. Although the role of PI3Kγ in inflammation and in cardiac function has been extensively investigated, the exploration of its CNS function is just at dawning. On this issue, it has been recently shown that the lack of PI3Kγ in mice leads to an impairment in synaptic plasticity associated with alterations suggestive of a role for PI3Kγ in mediating behavioral flexibility. Interestingly, among the neuropsychiatric disorders, the syndrome called ADHD (attention-deficit/hyperactivity disorder) involves difficulties in suppressing aggressive behaviors and displays many traits ascribable to behavioral inflexibility.

**Results**
Here, we show that the absence of PI3Kγ in mice reproduces the principal behavioral features observed in patients with ADHD. In particular, PI3Kγ KO mice exhibit deficits in the attentive and mnemonic domains, coupled with typical hyperactivity, as well as social dysfunctions. This behavioral phenotype is accompanied with neurochemical alterations, reflected by an unbalanced catecholaminergic activity in brain areas receiving projections from LC, where PI3Kγ is constitutively expressed. More importantly, we demonstrate, for the first time, that the observed behavioral phenotype, mediated by a kinase-independent mechanism, depends on a dysregulation of CREB signaling, exerted by PI3Kγ-PDE4D in LC, the largest cluster of noradrenergic neurons in the brain.

**Impact**
With our findings that strongly suggest PI3Kγ as a crucial player of ADHD, we propose an innovative molecular mechanism regulating behavioral flexibility in mouse models, which opens novel therapeutic perspectives and appropriate diagnostic strategies.

### Statistical analysis

Analyses were carried out using the SPSS 21.0 software (SPSS, Chicago, IL, USA). Unless otherwise indicated, results are presented as mean ± standard error of mean (SEM). All data normally distributed for more than two groups were examined by either one- or two-way ANOVA with repeated measures followed by Bonferroni's *post hoc* test. Unpaired two-tailed Student's *t*-test was used in case two groups were compared.

**Supplementary information** for this article is available online: http://embomolmed.embopress.org

## Acknowledgements

We thank Prof. Eric J. Nestler from Mount Sinai School of Medicine of New York, for kindly providing the AAV constructs used for the *in vivo* experiments of CREB inhibition. We thank Dr. Valeria Berardi for helping in the preparation of the manuscript. This work has been supported by Italian Ministry of Health RC to G.L. and D.C.

## Author contributions

ID performed behavioral phenotyping, analyzed and interpreted data, and drafted the manuscript. VF performed behavioral phenotyping, microsurgery and all the *in vivo* treatments. SF and RI performed molecular and histological analyses. FP performed microsurgery and all the *in vivo* treatments. AG and EH performed the analysis on PDE activity. AM performed statistical analysis. GL supervised the research, interpreted data, handled funding and critically read the manuscript. DC conceived the research, supervised experiments, analyzed and interpreted data, performed statistical analysis, handled funding and wrote the manuscript.

## Conflict of interest

The authors declare that they have no conflict of interest.

## Supporting Information

Supplementary Table S3

Review Process File

Source Data for Figure 3E
