# Supplementary figures and images for: Lack of kinase-independent activity of PI3Kγ in locus coeruleus induces ADHD symptoms through increased CREB signaling
Source: EMBO Mol Med. 2015 Apr 16;7(7):904–17. doi: 10.15252/emmm.201404697 (PMC4520656; doi:10.15252/emmm.201404697)

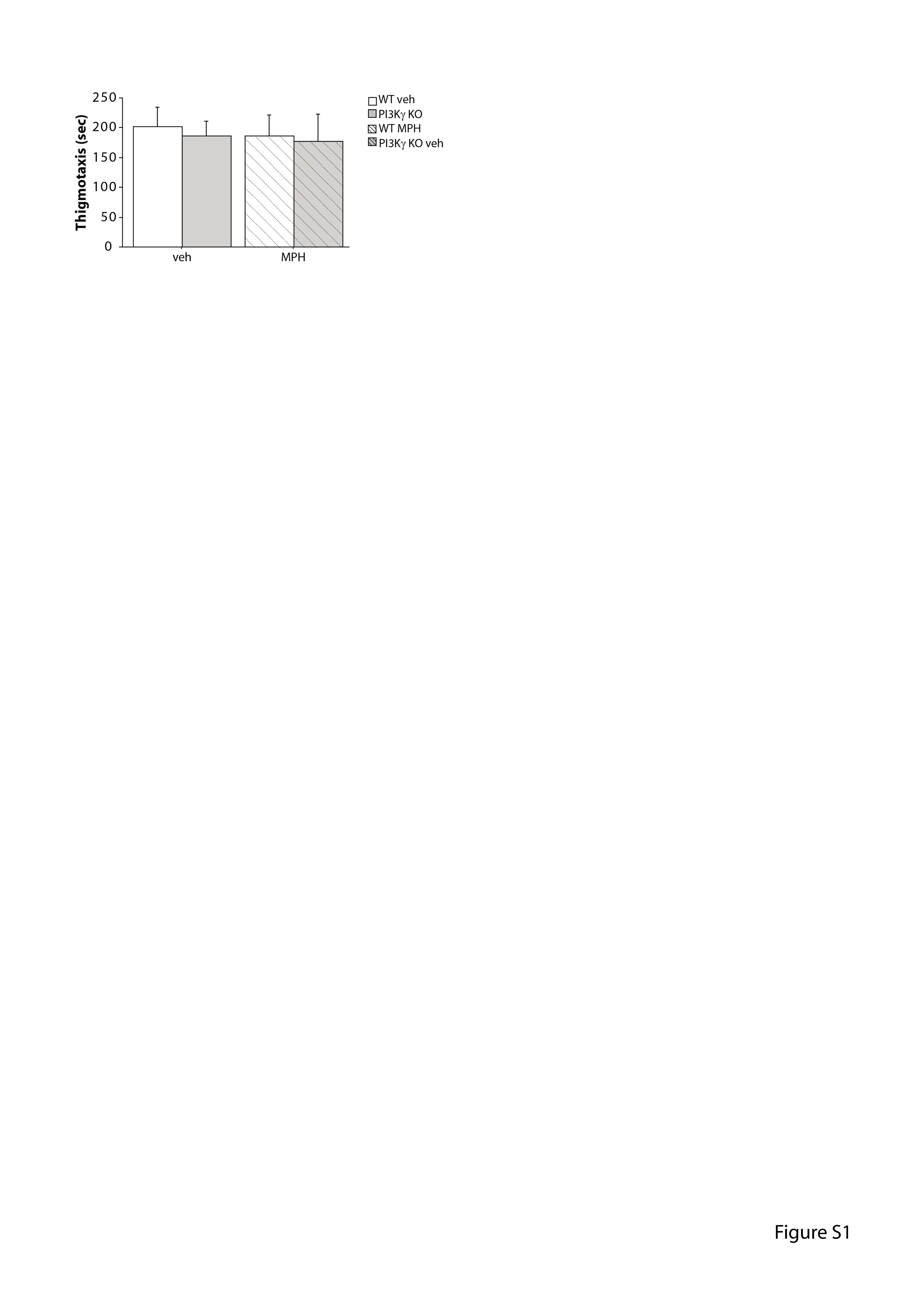

Supplement: Supplementary file 1 [file emmm0007-0904-sd1.tif]

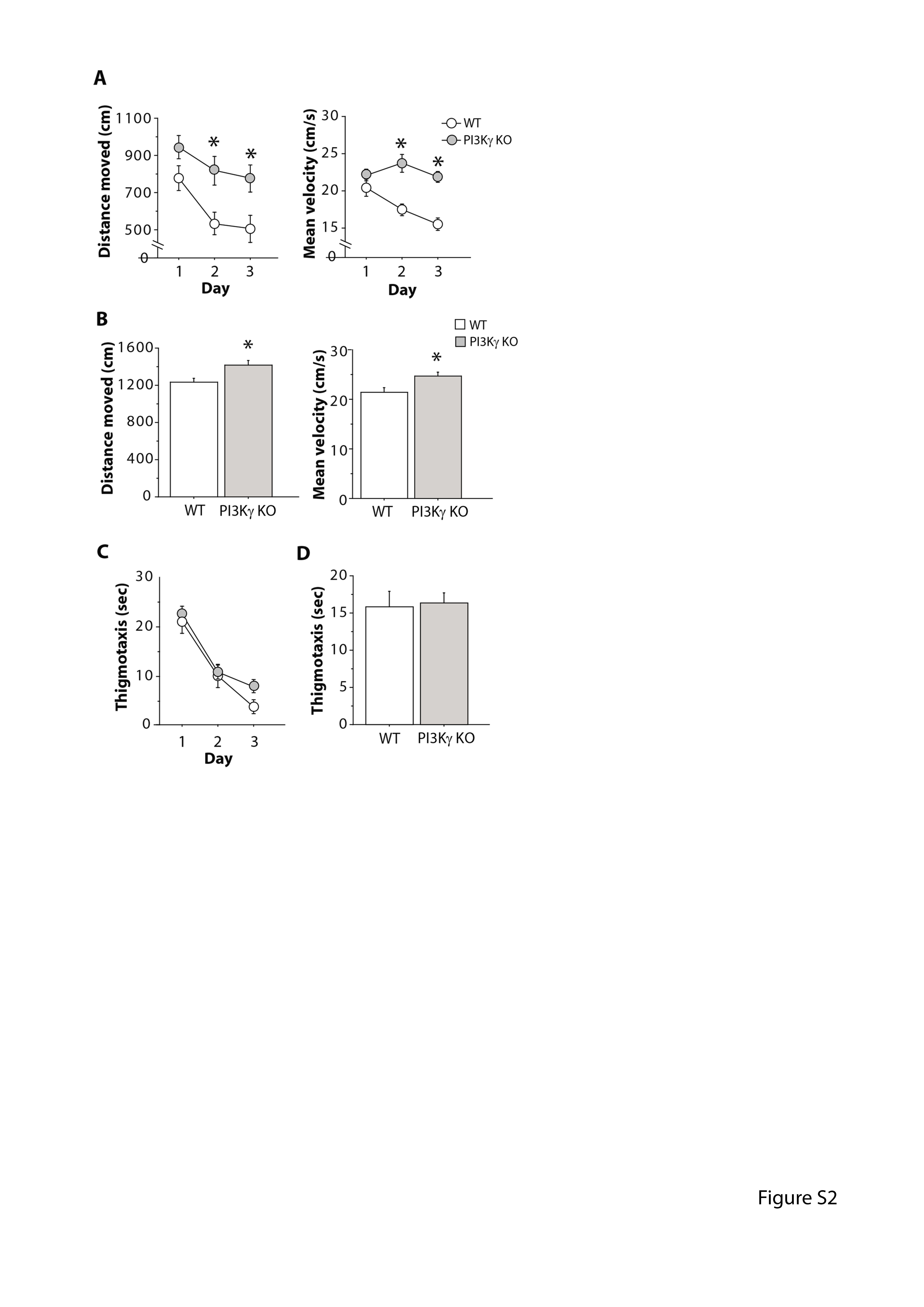

Supplement: Supplementary file 2 [file emmm0007-0904-sd2.tif]

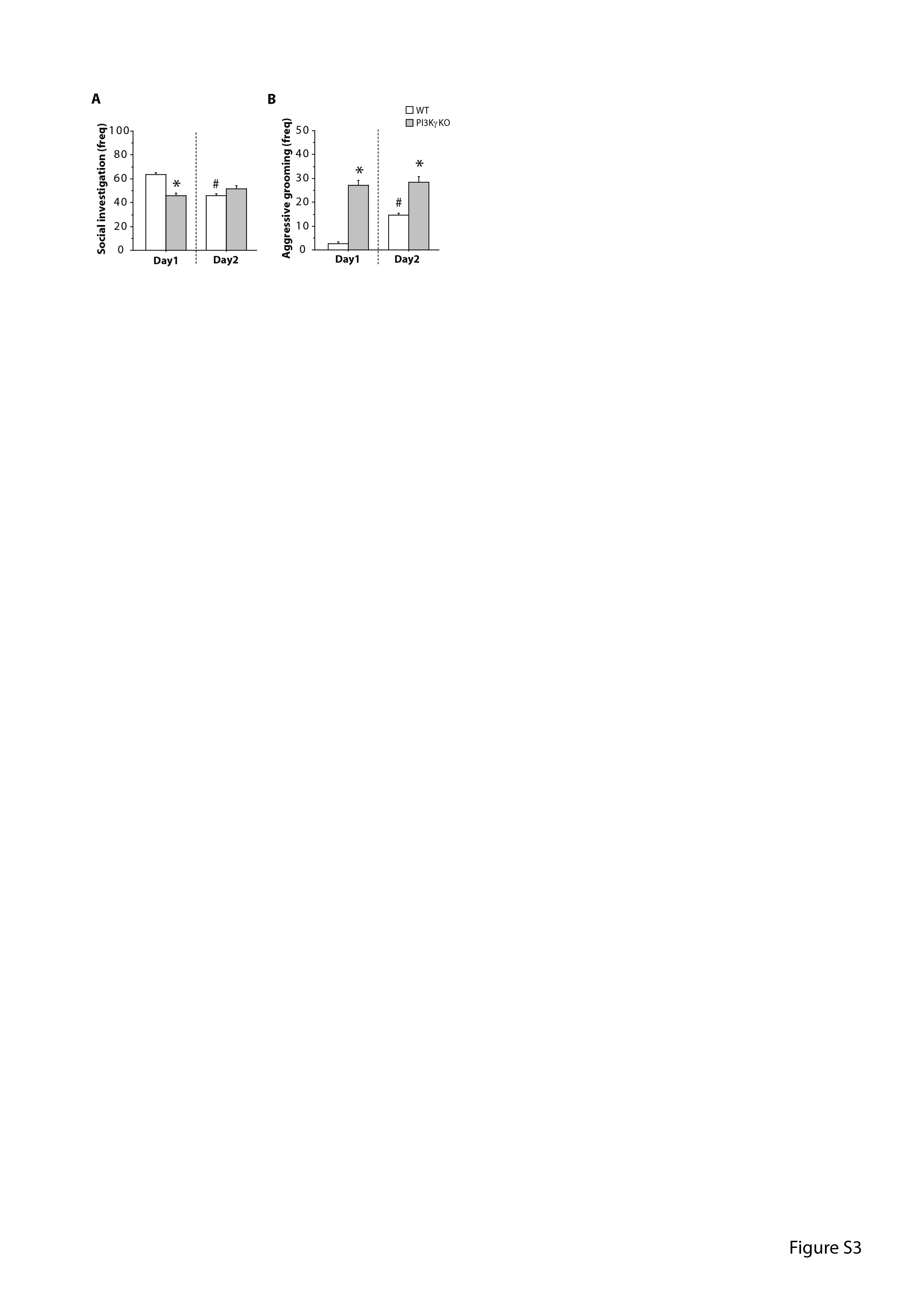

Supplement: Supplementary file 3 [file emmm0007-0904-sd3.tif]

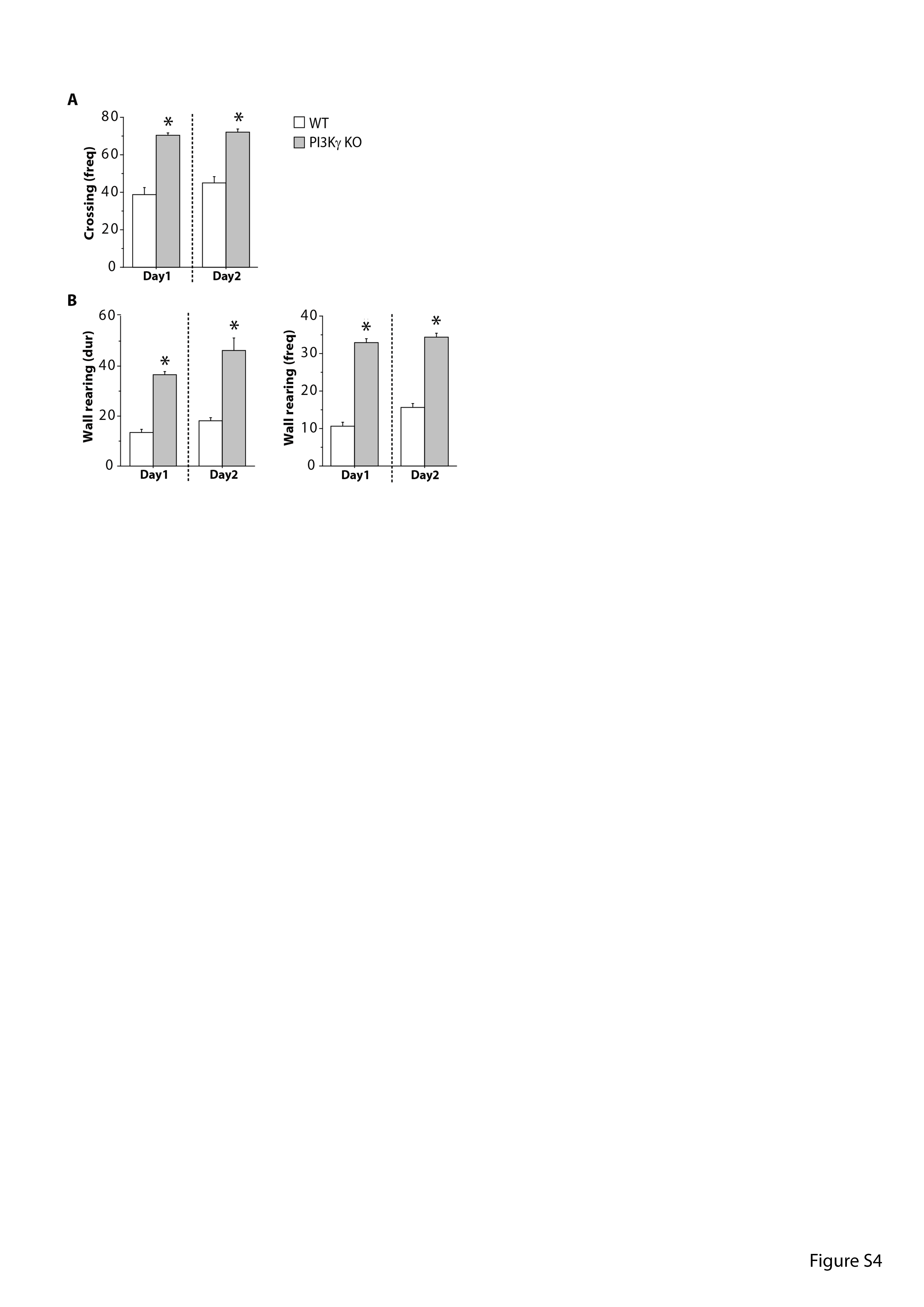

Supplement: Supplementary file 4 [file emmm0007-0904-sd4.tif]

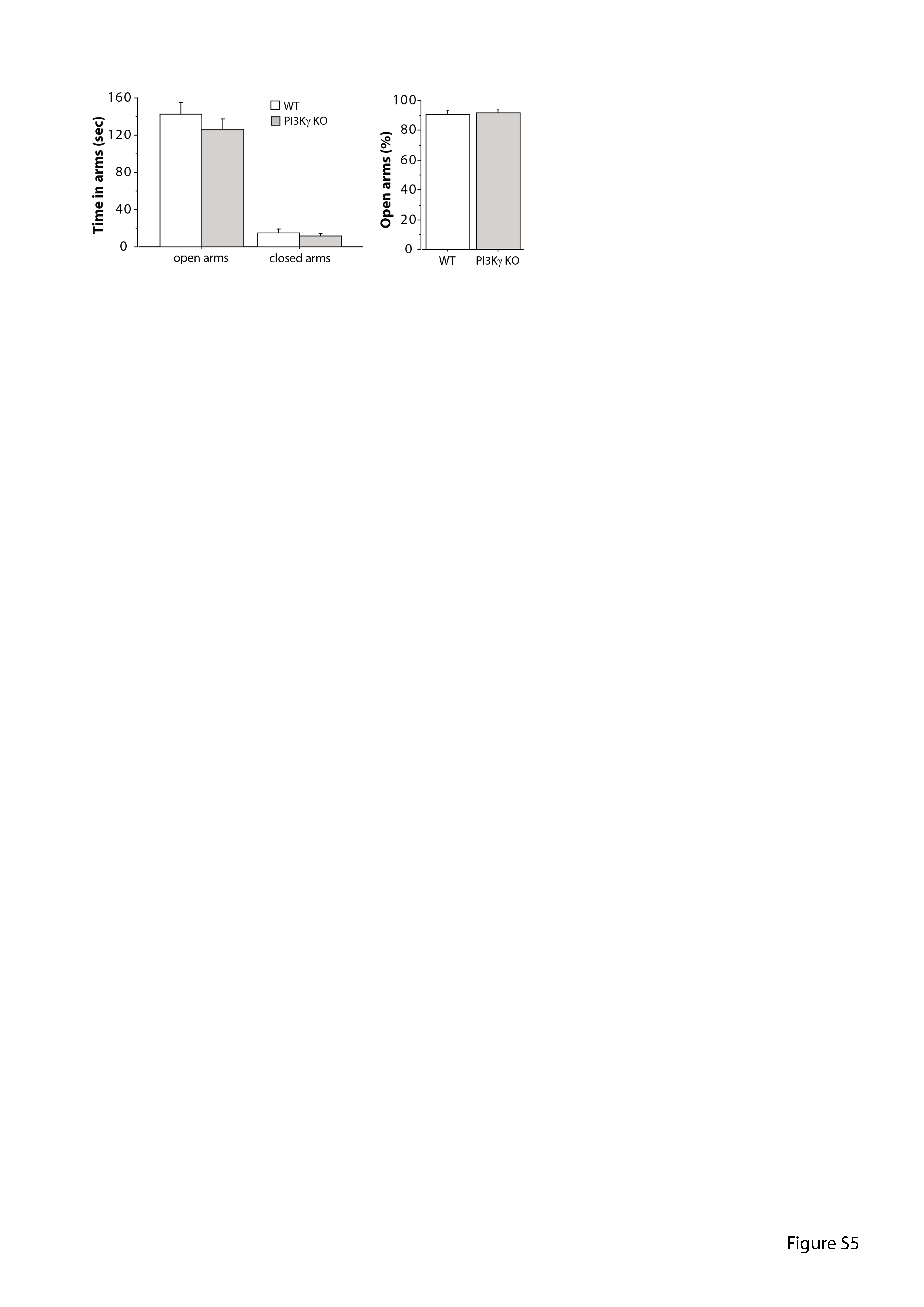

Supplement: Supplementary file 5 [file emmm0007-0904-sd5.tif]

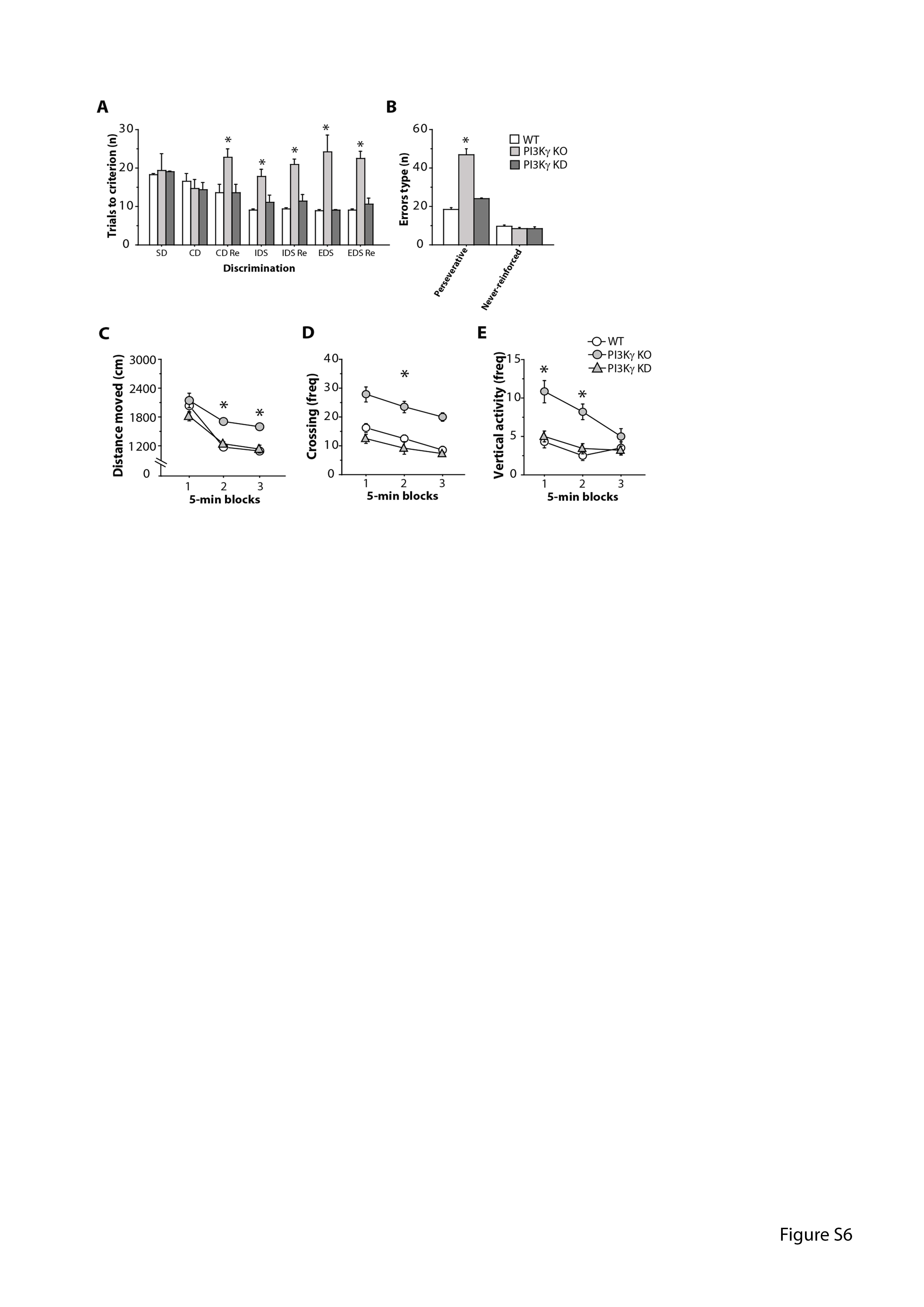

Supplement: Supplementary file 6 [file emmm0007-0904-sd6.tif]

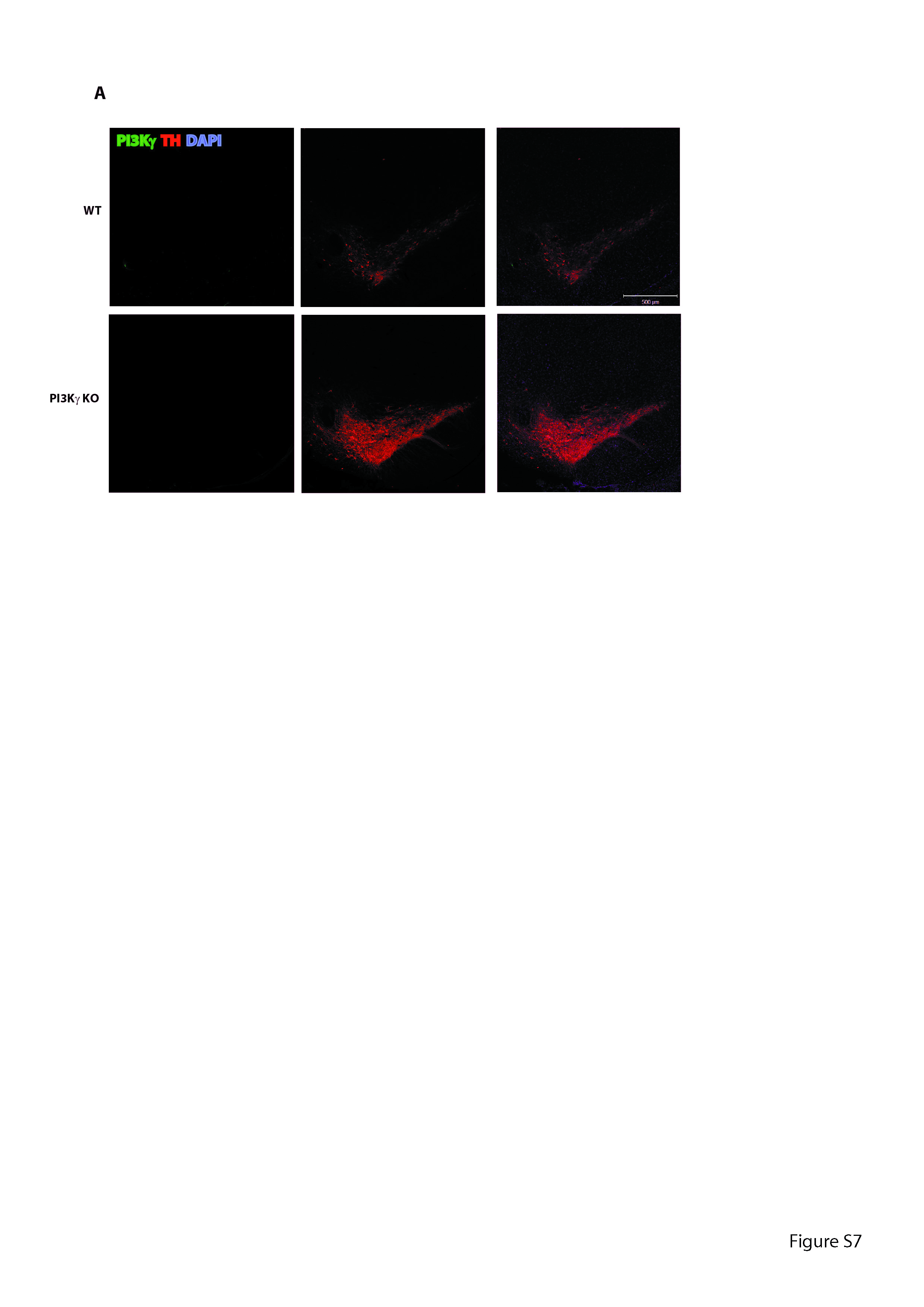

Supplement: Supplementary file 7 [file emmm0007-0904-sd7.tif]

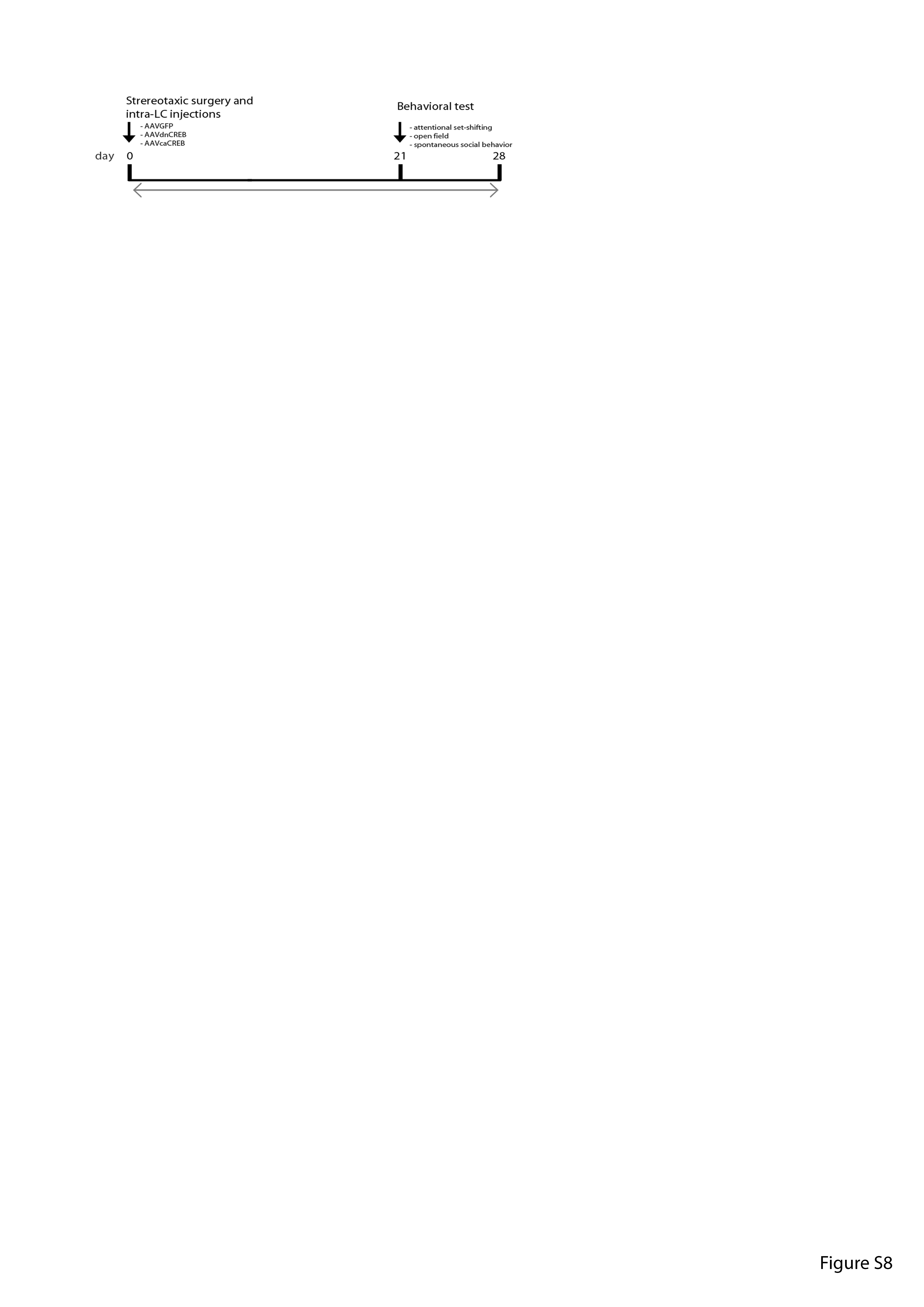

Supplement: Supplementary file 8 [file emmm0007-0904-sd8.tif]

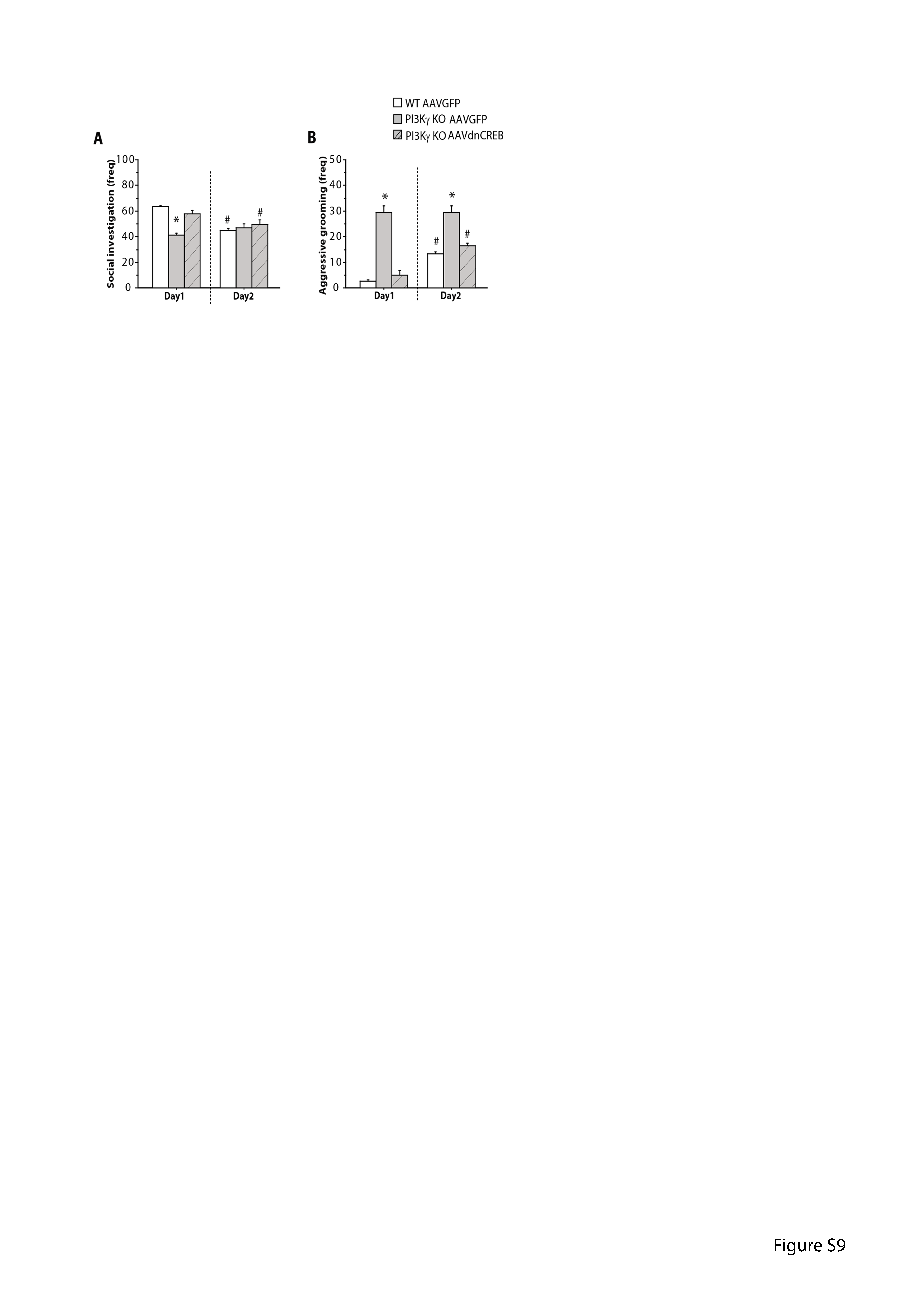

Supplement: Supplementary file 9 [file emmm0007-0904-sd9.tif]

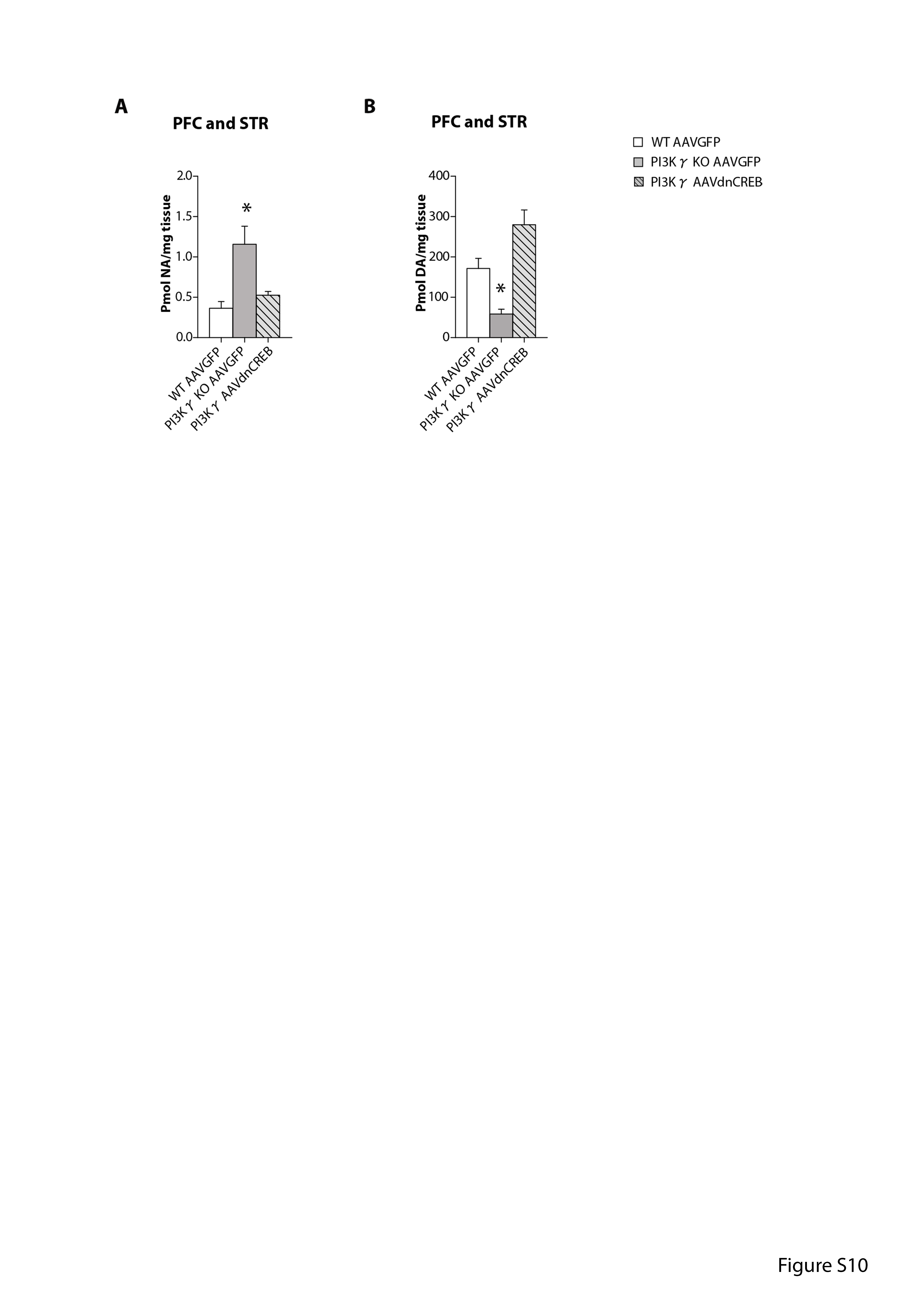

Supplement: Supplementary file 10 [file emmm0007-0904-sd10.tif]

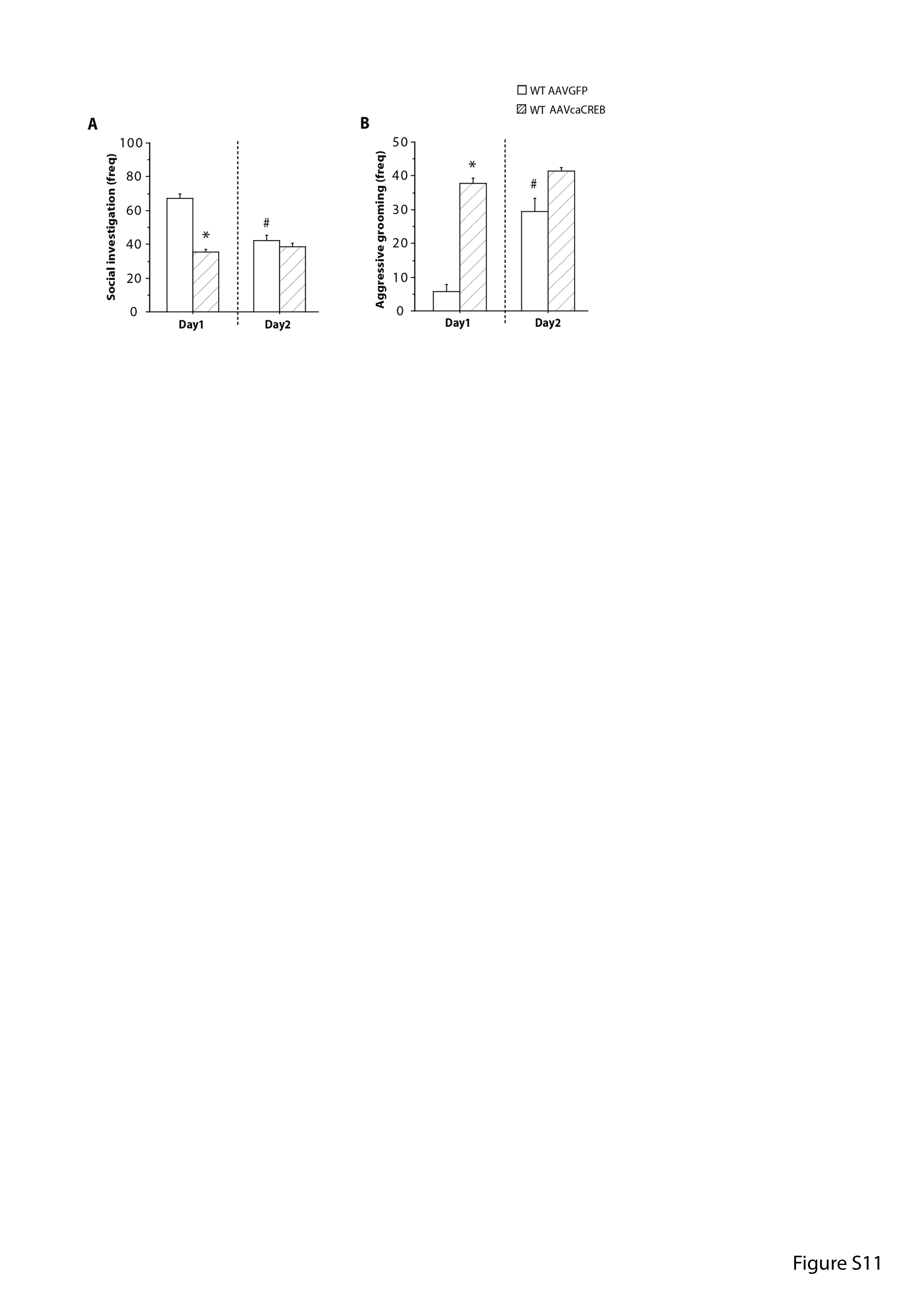

Supplement: Supplementary file 11 [file emmm0007-0904-sd11.tif]

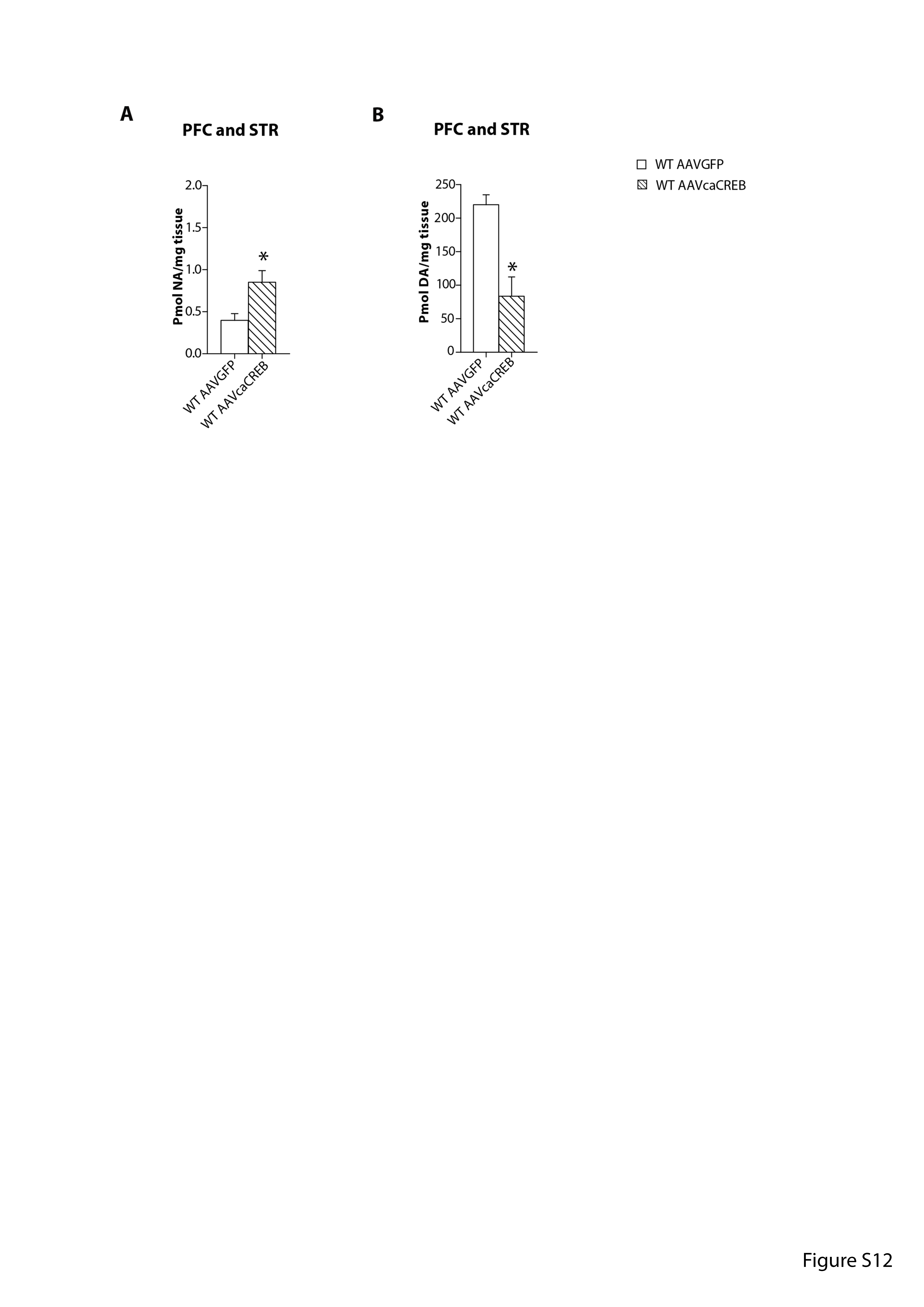

Supplement: Supplementary file 12 [file emmm0007-0904-sd12.tif]

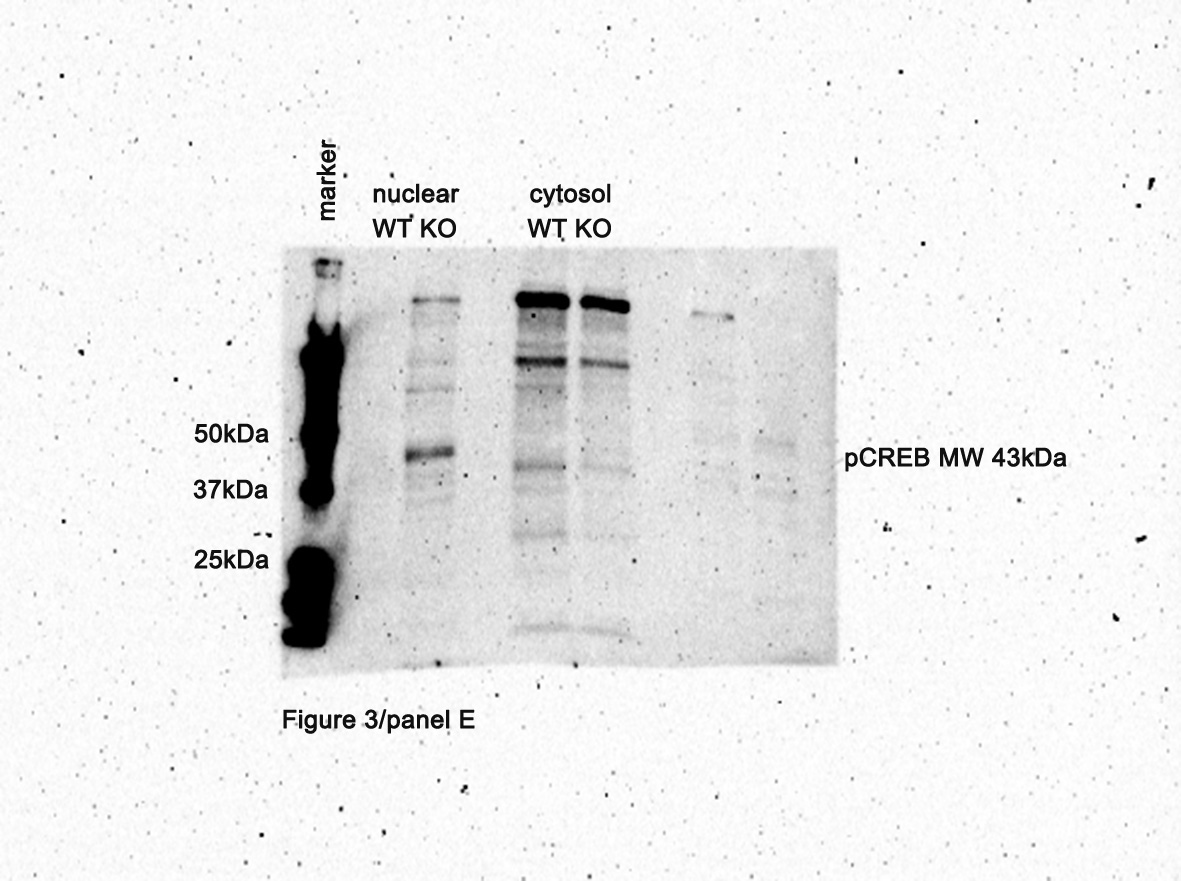

Supplement: Supplementary file 17 [file emmm0007-0904-sd17.jpg]
