# Supplementary material for: Lack of kinase-independent activity of PI3Kγ in locus coeruleus induces ADHD symptoms through increased CREB signaling
Source: EMBO Mol Med. 2015 Apr 16;7(7):904–17. doi: 10.15252/emmm.201404697 (PMC4520656; doi:10.15252/emmm.201404697)
Supplement: Supplementary file 13 [file emmm0007-0904-sd13.pdf]

Table S1. Summary of the major behavioral effects in the different experimental conditions

Experimental group	Behavioral test	Behavioral domain	Summary results
genotype treatment			
**PI3Kγ KO**	ASS<br>MWM	Cognition (i.e. attention, learning and memory)	Deficit in attention shifting and in memory track detection
	OF	Locomotor activity	Hyperactivity
	SSB	Social skills	Impaired social skills
**PI3Kγ KO MPH**	ASS<br>OF<br>SSB	Attention<br>Locomotor activity<br>Social skills	MPH treatment rescues from attentional deficit, hyperactivity and social dysfuctions
**PI3Kγ KD**	ASS<br>OF	Attention<br>Locomotor activity	ns, overlapped to WT<br>ns, overlapped to WT
**PI3Kγ KO dnCREB**	ASS<br>OF<br>SSB	Attention<br>Locomotor activity<br>Social skills	*In vivo* manipulation of CREB activity in LC, by AAV2 dnCREB injection, rescues from ADHD-like phenotype

MPH, Methylphenidate; ASS, Attentional set-shifting test; MWM, Morris Water Maze; OF, Opend Field; SSB, Spontaneous social behavior.
