# Supplementary material for: Lack of kinase-independent activity of PI3Kγ in locus coeruleus induces ADHD symptoms through increased CREB signaling
Source: EMBO Mol Med. 2015 Apr 16;7(7):904–17. doi: 10.15252/emmm.201404697 (PMC4520656; doi:10.15252/emmm.201404697)
Supplement: Supplementary file 14 [file emmm0007-0904-sd14.pdf]

Table S2. Stimulus used in the task

Digging media	Odour	Task
Sawdust	Cinnamon	SD and CD
Cotton	Sage	SD and CD
Shredded paper	oregano	IDS
Confetti	Thymus	IDS
Sand	Rosmary	EDS
Wood shavings	Cumin	EDS
