# Supplementary material for: Lack of kinase-independent activity of PI3Kγ in locus coeruleus induces ADHD symptoms through increased CREB signaling
Source: EMBO Mol Med. 2015 Apr 16;7(7):904–17. doi: 10.15252/emmm.201404697 (PMC4520656; doi:10.15252/emmm.201404697)
Supplement: Supplementary file 15 [file emmm0007-0904-sd15.pdf]

Table S3. Example of a complete attention set-shifting task

DIGGING MEDIA

Day	Stage	Rewarded stimuli	Non-rewarded stimuli
1	Simple discrimination -- SD	Sawdust	Cotton
2	Compound discrimination -- CD	Sawdust	Cotton
2	CD reversal -- CDR	Cotton	Sawdust
3	Intra-dimentional shift --IDS	Shredded paper	Confetti
3	IDS reversal --IDSR	Confetti	Shredded paper
4	Extra-dimensional shift --EDS	Rosmary	Cumin
4	EDS reversal -- EDSR	Cumin	Rosmary

ODOUR

Day	Stage	Rewarded stimuli	Non-rewarded stimuli
1	Simple discrimination -- SD	Cinnamon	Sage
2	Compound discrimination -- CD	Cinnamon	Sage
2	CD reversal -- CDR	Sage	Cinnamon
3	Intra-dimentional shift --IDS	Oregano	Thymus
3	IDS reversal --IDSR	Thymus	Oregano
4	Extra-dimensional shift --EDS	Wood shavings	Sand
4	EDS reversal -- EDSR	Sand	Wood shavings
