# Supplementary material for: Lack of kinase-independent activity of PI3Kγ in locus coeruleus induces ADHD symptoms through increased CREB signaling
Source: EMBO Mol Med. 2015 Apr 16;7(7):904–17. doi: 10.15252/emmm.201404697 (PMC4520656; doi:10.15252/emmm.201404697)
Supplement: Supplementary file 16 [file emmm0007-0904-sd16.pdf]

Lack of Kinase independent activity of PI3Kγ in locus coeruleus induces ADHD symptoms through increased CREB signaling

Ivana D'Andrea, Valentina Fardella, Stefania Fardella, Fabio Pallante, Alessandra Ghigo, Roberta Iacobucci, Angelo Maffei, Emilio Hirsch, Giuseppe Lembo and Daniela Carnevale

Corresponding authors: Giuseppe Lembo and Daniela Carnevale, IRCCS Neuromed

Review timeline:

Submission date:	30 September 2014
Editorial Decision:	03 November 2014
Revision received:	30 January 2015
Editorial Decision:	04 March 2015
Revision received:	16 March 2015
Accepted:	19 March 2015

Transaction Report:

(Note: With the exception of the correction of typographical or spelling errors that could be a source of ambiguity, letters and reports are not edited. The original formatting of letters and referee reports may not be reflected in this compilation.)

Editor: Céline Carret

1st Editorial Decision 03 November 2014

Thank you for the submission of your manuscript to EMBO Molecular Medicine. We have now heard back from the three referees whom we asked to evaluate your manuscript.

As you will see, while all three referees find the study interesting, they also recommend additional controls and clarifications/discussions to further increase the significance of the findings. As we believe that performing these additional controls would greatly improve the manuscript, we would like to encourage you to address all issues and resubmit your revised paper within a 3-months window.

I look forward to receiving your revised manuscript.

***** Reviewer's comments *****

Referee #1 (Remarks):

Authors focused on the role of PI3Kγ in the locus coeruleus, showing attention deficits and hyperactivity of PI3Kγ knock-out mice. Furthermore, they showed that increased CREB activity in locus coerules via a kinase-independent activity of PI3Kγ, inducing the changes of cathecolaminergic activity in projection areas, PFC and striatum. This is very interesting finding implying the new role of PI3Kγ in specific brain area, but there are some major concerns as following.

Major points:

© EMBO

1. This paper shows that PI3Kgamma KO mice have ADHD symptoms which they insist is due to altered CREB signaling in locus coeruleus(LC). However, they cannot insist that ADHD behavior, in general, is dependent on PI3Kgamma in the locus coeruleus. It may be one of many causes that results in ADHD symptoms, and in many other cases with ADHD symptoms, the PI3Kgamma activity may be normal in the locus coeruleus. The title should be, for example, 'Lack of Kinase independent activity of PI3Kgamma in locus coeruleus induces ADHD symptoms through increased CREB signaling'.
2. The paper strongly depends on the dominant negative CREB (dnCREB) overexpression experiments to link the altered CREB signaling in LC to the ADHD symptoms. For a better rescue experiment, they should show that dnCREB overexpression in LC doesn't have any effect on wildtype. To be more convincing, they may also overexpress CREB in LC to show that increased CREB signaling in LC is sufficient to induce ADHD symptoms.
3. There are no data for total distance moved in open field task (Fig. 1). I believe the increase of total distance moved in PI3Kγ KO is crucial to insist the hyperactive phenotype of PI3Kγ KO mice. Do PI3Kγ KO mice show increased total distance moved in open-field task?
4. Two clear phenotypes in open field task, number of crossing and vertical activity, seem to be more related with anxiety (Fig. 1D and 1E). In this point of view, PI3Kγ KO mice showed the lower anxiety level compared to control mice. I suggest to perform other anxiety-related tasks, for example, elevated-plus maze task.
5. Kim et al. (2011) also performed open field test using PI3Kγ KO mice, but there were no differences in total distance moved, velocity and anxiety (Supplementary fig. 5 in Kim et al.). What would be the explanation for these different results?

Minor comments:

1. What are the differences between Fig 2C, 2D and Fig E3A, E3B?
2. What does NA stand for?
3. There is mislabeled reference in Materials and methods.
"...mouse anti-PI3Kγ produced as previously described18,20"

Referee #2 (Comments on Novelty/Model System):

The manuscript provides a series of interesting and novel observations on PI3Kγ knockout (KO) mice. The main problem is whether their observations can be ascribed to the noradrenrgic system and whether the suggested cellular mechanism is correct.

Referee #2 (Remarks):

The present manuscript investigates PI3Kγ knockout (KO) mice in relation to ADHD. First, the authors show evidence for expression of the kinase in noradrenergic neurons of the Locus Coeruleus (LC). Second, they demonstrate behavioral deficits in the KO mice including hyperactivity and poor ability in set-shifting attention, and that these deficits are reversed by methylphenidate, a drug used to treat ADHD. The KO mice also show impaired memory and social skills. Third, the authors assess mice expressing a kinase-dead mutant (PI3Kγ KD) but surprisingly they cannot replicate the behavioral phenotype that was observed in the KO mice suggesting a kinase-independent mechanism. Fourth, the author show that PDE4D can be co-immunoprecipitated with PI3Kγ and that PDE4 activity is reduced in LC tissue. Fifth, the authors present evidence for an increased pCREB/CREB ratio as a presumed consequence of the reduced PDE4D activity. Finally, a dominant negative variant of CREB is expressed in LC of KO mice using an adeno-associated virus construct. This results in reversal of hyperactivity and attention deficits.

The manuscript describes a series of interesting observations relating to an ADHD-like phenotype of PI3Kγ KO mice. My main concern is the use of a global KO mouse combined with a sole focus on the noradrenergic system. PI3Kγ is indeed expressed in noradrenergic neurons but what about other neurons in other parts of the brain. Especially, expression in dopaminergic neurons could be of interest. Indeed the authors find evidence for altered dopaminergic homeostasis (decreased tissue DA). The authors state that this is due to an inhibitory effect of LC on dopaminergic transmission

but this statement is truly hand waving and not supported by any data. It is also important to note that MPH is BOTH a DAT and NET inhibitor, thus, reversal of the phenotype can both relate to the dopamine and the noradrenergic system. I acknowledge that expression of a dominant negative CREB (dnCREB) in LC reverses hyperactivity and attention deficits in the KO mice but a potential effect of dnCREB on WT mice is not presented. Also, although PI3Kγ may regulate PDE4 activity, expression of a dnCREB does not directly assess the importance of the kinase, which is what the authors want to conclude about. Ideally, a KO of PI3Kγ selectively in noradrenergic neurons is required if the authors want to conclude as they do. Otherwise, conclusions throughout the manuscript should be substantially moderated. The issue must also be carefully and thoroughly discussed in the discussion. Another possibility is to more specifically assess involvement of the dopamine system in the observed phenotype.

My second major concern is the cellular mechanisms underlying the presented observations. Indeed it is possible that PI3Kγ could regulate PDE4 directly in a kinase independent fashion but no evidence is shown for this hypothesis. The authors only show a moderate though significant reduction in PDE4D activity in tissue from the KO mice. In principle, there could be many direct or indirect reasons for this reduction. The co-immunoprecipitation just shows that the kinase and PDEs are part of the same complex. It should be possible to design in vitro experiments to biochemically demonstrate that PI3Kγ can regulate PDE activity in a kinase-independent manner.

Referee #3 (Comments on Novelty/Model System):

The mouse model is best suited for this study

Referee #3 (Remarks):

This excellent study uses genetic mouse models to demonstrate an essential role for PI3Kγ signaling in a set of behavioral responses, which altogether model main features of attention deficit/hyperactivity disorders (ADHD). The authors first find that locomotor activity, attention, memory and sociability are altered in PI3Kγ knockout mice, using dedicated testing for ADHD-like behaviors, and show that these deficits are reversed by methylphenidate (MPH). Further investigating downstream signaling pathways, the authors show lack of behavioral deficits in knock-in mice lacking PI3Kγ kinase activity, modified PDE4D, noradrenaline and dopamine in several brain areas, and increased pCREB in the locus coeruleus (LC). Virally mediated overexpression of a dominant negative form of CREB in the LC finally reverses behavioral phenotypes of PI3Kγ knockout mice. Together, the data strongly suggests that PI3Kγ regulates ADHD-like behaviors via a kinase-independent but CREB-dependent mechanism in noradrenergic neurons of the locus.
The manuscript is very well written, and is accessible for a general audience. The study is innovative, since PI3Kγ function has been barely studied in the brain. The experimental flow is based on strong rationales and leads to identify an entire signaling pathway that potentially underlies ADHD-like behaviors; the study uses appropriate genetic tools and is conducted highly rigorously; behavioral data are convincing and support the author's conclusions; finally the work provides a novel mouse model for ADHD, which represents a true progress in a field where animal models are particularly scarce. Altogether, this is a high-quality manuscript that will be of broad interest for the readership of EMBO Molecular Medicine.

Here are a number of minor comments or suggestions that should improve the manuscript:

1.The authors indicate strong and selective PI3Kγ expression in the LC, an observation that forms a rationale for the study. Is PI3Kγ absent from the rest of the brain?
2. MPH efficiently reverses behavioral deficits of PI3Kγ knockout mice in the ASS assay. Does MPH also reverse memory performance in the WM, social interaction deficits, and/or altered DA and NA levels in the knockout mice? Although not essential for the study, such data would strengthen the notion that PI3Kγ knockout mice represent an excellent new genetic mouse model of ADHD.
3. Based on measures of bioamine levels, authors speculate on increased NA activity and possible inhibition of DA transmission. Data, however, only show modifications of tissue NA and DA levels in cortex and striatum, respectively. Conclusions on neurotransmission seem premature, and further,

the potential interaction between NA in the cortex and DA in the striatum, in this case, may be clarified.
4. AAV-CREBdn experiment: did the authors verify for correct injection site and CREBdn overexpression for each individual animal, before completing behavioral statistical analyses?
5. The statement that "balanced cAMP-CREB signaling pathway is well known to be needed for proper working of the LC" (discussion) is a bit light, and some references may be useful for the reader.
6. Has any PI3Kγ gene variant been described in humans, potentially associated with ADHD?
7. Mouse genetic background (method section): it seems that several genetic backgrounds were used throughout study, with a tentative explanation for rationale and strain comparison. Please clarify this part.
8. Labels for antibodies used in immunohistochemical experiments in Fig 1A (and other similar figures) are not visible. Please improve.

1st Revision - authors' response 30 January 2015

Referee #1 (Remarks):

Authors focused on the role of PI3Kγ in the locus coeruleus, showing attention deficits and hyperactivity of PI3Kg knock-out mice. Furthermore, they showed that increased CREB activity in locus coerules via a kinase-independent activity of PI3Kg inducing the changes of cathecolaminergic activity in projection areas, PFC and striatum. This is very interesting finding implying the new role of PI3Kg in specific brain area, but there are some major concerns as following.

Major points:

1. This paper shows that PI3Kgamma KO mice have ADHD symptoms which they insist is due to altered CREB signaling in locus coeruleus (LC). However, they cannot insist that ADHD behavior, in general, is dependent on PI3Kgamma in the locus coeruleus. It may be one of many causes that results in ADHD symptoms, and in many other cases with ADHD symptoms, the PI3Kgamma activity may be normal in the locus coeruleus. The title should be, for example, 'Lack of Kinase independent activity of PI3Kgamma in locus coeruleus induces ADHD symptoms through increased CREB signaling'.

We thank the Reviewer for her/his suggestion. We are aware that we cannot conclude that, in general, ADHD is dependent on PI3Kg in the locus coeruleus and we have accordingly modified the title.

2. The paper strongly depends on the dominant negative CREB (dnCREB) overexpression experiments to link the altered CREB signaling in LC to the ADHD symptoms. For a better rescue experiment, they should show that dnCREB overexpression in LC doesn't have any effect on wildtype. To be more convincing, they may also overexpress CREB in LC to show that increased CREB signaling in LC is sufficient to induce ADHD symptoms.

As regard this issue we have two main points:

1) we did not use the dnCREB overexrpressor in WT mice since it has been previously demonstrated by Dr. Nestler group that the overexpression of a dnCREB in WT locus coeruleus, has profound effects on the activity of noradrenergic LC neurons (Cao et al, PNAS 2010). In particular, it has been shown that dnCREB significantly lowers the firing rate of LC neurons, thus hampering its normal functions. Accordingly, we did not use the AAV dnCREB in WT mice, being aware that we already know that it has negative effects in WT mice.

2) As suggested by the Reviewer, we performed an experiment by using a constitutively active CREB (caCREB) overexpressed by AAV in the LC of WT mice. As shown in the revised manuscript (Figure 5 and E11-12) we induced in WT mice a phenotype resembling ADHD symptoms and overlapping the one of PI3Kg KO mice. The novel data further support our mechanistic conclusion on the role of CREB in LC.

3. There are no data for total distance moved in open field task (Fig. 1). I believe the increase of total distance moved in PI3Kg KO is crucial to insist the hyperactive phenotype of PI3Kg KO mice. Do PI3Kg KO mice show increased total distance moved in open-field task?

As suggested by Reviewer, we have now provided the data of total distance moved for all experiments as shown in the revised version of the manuscript (Figure 1D, 4D and 5C). In agreement to the other locomotor parameters analyzed, PI3Kγ KO mice show increased distance moved as compared to WT mice, confirming their hyperactive phenotype.

4. Two clear phenotypes in open field task, number of crossing and vertical activity, seem to be more related with anxiety (Fig. 1D and 1E). In this point of view, PI3Kg KO mice showed the lower anxiety level compared to control mice. I suggest to perform other anxiety-related tasks, for example, elevated-plus maze task.

In order to address the Reviewer's request and exclude that the hyperactive phenotype could be related to anxiety, we performed the elevated plus maze test, assessing the anxiety-like behavior in PI3Kγ KO and WT mice. As shown in the revised version of the manuscript, no difference emerged by data between KO and WT mice (Figure E5). Furthermore, we have now added in the supplementary figures (Figure E1) the thigmotaxis behavior as evaluated in the open field (i.e. tendency to spend more time closer the wall, considered as index of anxiety-like behavior), further confirming no difference in anxiety-related response. Thus, as explained in the text we can now conclude that the hyperactive phenotype displayed by PI3Kg KO mice is not affected by anxiety like behaviors.

5. Kim et al. (2011) also performed open field test using PI3Kg KO mice, but there were no differences in total distance moved, velocity and anxiety (Supplementary fig. 5 in Kim et al.). What would be the explanation for these different results?

We thank the Reviewer for this observation that, at first, surprised us as well. However, at a deeper analysis of the results from that paper, we get that the experimental paradigm exploited by Kim and colleagues, uses a white open field apparatus. It has been demonstrated that bright light induces anxiety in mice and increases motility parameters, taking advantage of their naturally light-aversion (Bourin M, Hascoet M. The mouse light/dark box test. Eur J Pharmacol. 2003; 463(1–3):55–65). Indeed, in the study of Kim and colleagues, the distance moved values, appear closer to hyperactivity than the physiological locomotor levels commonly observed, thus suggesting a possible ceiling effect on WT mice, induced by the experimental setting itself. As regard the anxiety response, our new results provided from Elevated Plus Maze test, confirm data from Kim and colleagues.

Minor comments:

1. What are the differences between Fig 2C, 2D and Fig E3A, E3B?

We apologize for the description that maybe is not sufficiently clear. This information has been now clarified in the text, "The behavioral categories and elements scored for both frequency and duration were: social investigation (sniffing and grooming the partner in all body regions indicative of affiliative behavior) and aggressive grooming (violent grooming of the animal on the back of the partner)". This information has been also added in the figure legends.

2. What does NA stand for?

We have now checked the manuscript and spelled it out at first mention (NA stands for noradrenalin).

3. There is mislabeled reference in Materials and methods. "...mouse anti-PI3Kg produced as previously described18,20"

We thank the Reviewer for noticing this discrepancy and we apologize for it. We have now accordingly changed the references.

Referee #2 (Comments on Novelty/Model System):

The manuscript provides a series of interesting and novel observations on PI3Kg knockout (KO) mice. The main problem is whether their observations can be ascribed to the noradrenrgic system and whether the suggested cellular mechanism is correct.

Referee #2 (Remarks):

The present manuscript investigates PI3Kg knockout (KO) mice in relation to ADHD. First, the authors show evidence for expression of the kinase in noradrenergic neurons of the Locus Coeruleus (LC). Second, they demonstrate behavioral deficits in the KO mice including hyperactivity and poor ability in set-shifting attention, and that these deficits are reversed by methylphenidate, a drug used to treat ADHD. The KO mice also show impaired memory and social skills. Third, the authors assess mice expressing a kinase-dead mutant (PI3Kg KD) but surprisingly they cannot replicate the behavioral phenotype that was observed in the KO mice suggesting a kinase-independent mechanism. Fourth, the author show that PDE4D can be co-immunoprecipitated with PI3Kg and that PDE4 activity is reduced in LC tissue. Fifth, the authors present evidence for an increased pCREB/CREB ratio as a presumed consequence of the reduced PDE4D activity. Finally, a dominant negative variant of CREB is expressed in LC of KO mice using an adeno-associated virus construct. This results in reversal of hyperactivity and attention deficits.

The manuscript describes a series of interesting observations relating to an ADHD-like phenotype of PI3Kg KO mice. My main concern is the use of a global KO mouse combined with a sole focus on the noradrenergic system. PI3Kg is indeed expressed in noradrenergic neurons but what about other neurons in other parts of the brain. Especially, expression in dopaminergic neurons could be of interest. Indeed the authors find evidence for altered dopaminergic homeostasis (decreased tissue DA). The authors state that this is due to an inhibitory effect of LC on dopaminergic transmission but this statement is truly hand waving and not supported by any data. It is also important to note that MPH is BOTH a DAT and NET inhibitor, thus, reversal of the phenotype can both relate to the dopamine and the noradrenergic system. I acknowledge that expression of a dominant negative CREB (dnCREB) in LC reverses hyperactivity and attention deficits in the KO mice but a potential effect of dnCREB on WT mice is not presented. Also, although PI3Kg may regulate PDE4 activity, expression of a dnCREB does not directly assess the importance of the kinase, which is what the authors want to conclude about. Ideally, a KO of PI3Kg selectively in noradrenergic neurons is required if the authors want to conclude as they do. Otherwise, conclusions throughout the manuscript should be substantially moderated. The issue must also be carefully and thoroughly discussed in the discussion. Another possibility is to more specifically assess involvement of the dopamine system in the observed phenotype.

We thank the Reviewer for her/his observations.

1) As regard the focus that we made in the LC coeruleus, this conclusion has some explanations. It is correct that PI3Kg is expressed also elsewhere in the brain. Indeed, Kim et al (Nat Med 2011) showed in their paper expression of the mRNA in brain areas (cortex and hippocampus) related to synaptic functions and we have recently published a paper

where we explored the role of PI3Kg in hypothalamic neurons (Perino et al, Science Signaling 2014). However, these brain areas would be improbable candidate for a role in ADHD like behaviors. Instead, the concern of the Reviewer about the possibility that PI3Kg could be expressed in dopaminergic neurons is correct and indeed, we looked for that, finding no positivity for PI3Kg in both ventral tegmental area (VTA) and substantia nigra (SN). In the previous version of the manuscript, we omitted this negative result. However, accordingly to the Reviewer's suggestion we have now enclosed a novel figure (Figure E7) clearly showing that PI3Kg is absent in VTA/SN and thus further supporting the conclusion that PI3Kg play a role in the noradrenergic LC neurons to control ADHD like behaviors. On this issue, it is also worth noting that the phenotype rescue experiment with AAV overexpressing dnCREB, selectively injected in the LC of PI3Kg KO mice, is another point that strengthens our conclusion on the role of PI3Kg in the LC. Indeed, if PI3Kg had played other roles in different areas, we would have expected no rescue or partial effect by a selective correction of CREB activity in the LC. Moreover, we have now added novel data about the impact of the AAV dnCREB injected in the LC on PFC and STR dopamine/noradrenalin. In particular, we have found that, when we overexpressed a dnCREB in the LC of PI3Kg KO mice, the levels of DA/NA in PFC and STR returned to levels comparable to that of WT mice (Figure E10). Conversely, when we overexpressed a constitutively active CREB in the LC of WT mice, we obtained a dysregulation of DA/NA levels, comparable to that of PI3Kg KO mice (Figure E12).

2) The experiment with MPH has been performed in order to demonstrate the validity with the human pathology, and not a mechanistic explanation of the phenotype.
3) Concerning the lack of an experimental group testing the effect of the overexpression of dnCREB in WT mice, please refer to the response to point 2 of Reviewer #1.
4) We agree with the Reviewer that the experiment exploiting the dnCREB does not directly address the mechanism linking PI3Kg to PDE4. However, it is well known that PDE activity is needed to keep controlled levels of cAMP and that, this latter is a crucial regulator of LC activity by determining the activation levels of its response element, CREB. Thus, we have targeted the level of activation of CREB in our mechanistic experiments. By this way, we have proved that PI3Kg, its scaffold activity, specifically controls the levels of CREB activity in the LC and that, in turn, this is necessary for the regulation of behavioral responses that, when altered, give rise to ADHD like phenotypes. However, we have also carefully revised the manuscript and moderated the conclusions.

My second major concern is the cellular mechanisms underlying the presented observations. Indeed it is possible that PI3Kg could regulate PDE4 directly in a kinase independent fashion but no evidence is shown for this hypothesis. The authors only show a moderate though significant reduction in PDE4D activity in tissue from the KO mice. In principle, there could be many direct or indirect reasons for this reduction. The co-immunoprecipitation just shows that the kinase and PDEs are part of the same complex. It should be possible to design in vitro experiments to biochemically demonstrate that PI3Kg can regulate PDE activity in a kinase-independent manner.

We understand this issue and we would like to highlight the large body of evidence pointing to a specific role of PI3Kg scaffold function in the control of cAMP levels (Patrucco Cell 2004, Perino Molecular Cell 2011, Ghigo Circulation 2012, Schmidt Neuroscience 2013). The observation that different phenotypes found in PI3Kg kinase-dead knock-in (KD) and knock-out (KO) mice has been the key to distinguish the scaffold function from the involvement of the classical PI3Kg activation, through the PIP3/Akt pathway. In order to dissect the contribution of PI3Kg kinase-dependent and -independent effects in the ADHD like phenotype, we now compared KD and KO mice in the experiment assessing the PDE4D activity. Our novel results reported in the revised version of the manuscript show reduced PDE4D activity in KO LC, but normal PDE4D activity in KD LC (Figure 3A). This difference conclusively demonstrates the involvement in the ADHD like phenotype of the scaffold function only.

Referee #3 (Comments on Novelty/Model System):

The mouse model is best suited for this study

Referee #3 (Remarks):

This excellent study uses genetic mouse models to demonstrate an essential role for PI3Kg signaling in a set of behavioral responses, which altogether model main features of attention deficit/hyperactivity disorders (ADHD). The authors first find that locomotor activity, attention, memory and sociability are altered in PI3Kg knockout mice, using dedicated testing for ADHD-like behaviors, and show that these deficits are reversed by methylphenidate (MPH). Further investigating downstream signaling pathways, the authors show lack of behavioral deficits in knock-in mice lacking PI3Kg kinase activity PDE4D, noradrenaline and dopamine in several brain areas, and increased pCREB in the locus coeruleus (LC). Virally mediated overexpression of a dominant negative form of CREB in the LC finally reverses behavioral phenotypes of PI3Kg knockout mice. Together, the data strongly suggests that PI3Kg regulates ADHD-like behaviors via a kinase-independent but CREB-dependent mechanism in noradrenergic neurons of the locus.

The manuscript is very well written, and is accessible for a general audience. The study is innovative, since PI3Kg function has been barely studied in the brain. The experimental flow is based on strong rationales and leads to identify an entire signaling pathway that potentially underlies ADHD-like behaviors; the study uses appropriate genetic tools and is conducted highly rigorously; behavioral data are convincing and support the author's conclusions; finally the work provides a novel mouse model for ADHD, which represents a true progress in a field where animal models are particularly scarce. Altogether, this high-quality manuscript will be of broad interest for the readership of EMBO Molecular Medicine.

Here are a number of minor comments or suggestions that should improve the manuscript:

1. The authors indicate strong and selective PI3Kg expression in the LC, an observation that forms a rationale for the study. Is PI3Kg absent from the rest of the brain?

As stated in the response to Reviewer # 2 (point 1), PI3Kg is also expressed in other brain areas, in particular in the cortex and hippocampus (Kim et al, Nat Med). Moreover, we have recently found that it is also co-expressed with PI3Kb in the hypothalamus (Perino et al, Science Signaling 2014). However, the presence of PI3Kg in the brainstem, throughout the LC, made us candidate this enzyme for a role in regulating this noradrenergic nucleus. Moreover, we now report that PI3Kg is absent from the other main monoaminergic area, namely the VTA and SN (Figure E7), thus further supporting that the ADHD like behaviors are regulated by the presence of PI3Kg in the LC. However, we have revised the sentence at the beginning of results, considering that the word “selective” may be incorrect.

2. MPH efficiently reverses behavioral deficits of PI3Kg knockout mice in the ASS assay. Does MPH also reverse memory performance in the WM, social interaction deficits, and/or altered DA and NA levels in the knockout mice? Although not essential for the study, such data would strengthen the notion that PI3Kg knockout mice represent an excellent new genetic mouse model of ADHD.

When we planned the experiment with the MPH, we decided to test the therapeutic effect just in some of the key phenotypes resembling ADHD. Moreover, it should be noticed that it has been demonstrated that similar doses of MPH, although having no effect in locomotor and attentive functions of WT mice, are able to improve memory retention (Carmack et al, 2014), thus discouraging us to test it in the MWM. As regard the DA and NA levels, the expected effect of MPH is a nonspecific monoaminergic stimulant drug. Thus, we did not test whether this treatment would have an impact on the mechanism proposed for PI3Kg in controlling DA and NA levels through regulation of CREB activity in the LC.

3. Based on measures of bioamine levels, authors speculate on increased NA activity and possible inhibition of DA transmission. Data, however, only show modifications of tissue NA and DA levels

in cortex and striatum, respectively. Conclusions on neurotransmission seem premature, and further, the potential interaction between NA in the cortex and DA in the striatum, in this case, may be clarified.

The Reviewer's remark is correct and we have accordingly modified the conclusions about neurotransmission.

4. AAV-CREBdn experiment: did the authors verify for correct injection site and CREBdn overexpression for each individual animal, before completing behavioral statistical analyses?

As regard this point, we have checked the expression of dnCREB and caCREB for each animal. We have not excluded any mouse from each experimental group, since we get correct injection and virus expression for each mouse. Concerning the stereotaxic procedure itself, we have recorded a 60-70% success rate; however, when the animal recovered from intervention, we had good expression of AAV in all mice survived. This result has been obtained by an intensive training with a motorized stereotaxis instrument that allows great accuracy and higher throughput than conventional ones.

5. The statement that "balanced cAMP-CREB signaling pathway is well known to be needed for proper working of the LC" (discussion) is a bit light, and some references may be useful for the reader.

We thank the Reviewer for noticing that. We have accordingly reviewed the text and included references.

6. Has any PI3Kg gene variant been described in humans, potentially associated with ADHD?

We thank the Reviewer for raising this interesting matter. As regard, it has been reported a genetic link between PI3Kg dysfunction and mental disorders, particularly autism (Kratz et al, 2002; Serajee et al, 2003). It should be noticed that, for long time, autism and, more in general, autism spectrum disorders (ASD), have been considered somewhat completely distinct from ADHD. However, it is tempt this belief has been reevaluated when many observational data put in light a frequent co-occurrence of the two conditions, opening the possibility that some symptoms found in ASD meet also the criteria of ADHD.

7. Mouse genetic background (method section): it seems that several genetic backgrounds were used throughout study, with a tentative explanation for rationale and strain comparison. Please clarify this part.

The Reviewer correctly point to the variety of genetic background used in this study. The use of both C57Bl/6J and 129Sv/Pas backgrounds comes from the generation of different transgenic models. In particular, the PI3Kg KO was firstly generated in 129Sv/Pas background and then backcrossed in C57Bl/6J, whereas the PI3Kg KD has been generated in C57Bl/6J. As first, we explored the phenotype of PI3Kg KO mice in the 129Sv/Pas background, which is known to have better response to the behavioral characteristics investigated in this study. When we found the ADHD phenotype, we analyzed the PI3Kg KO mice in C57Bl/6J background, finding results overlapping to that observed in the 129Sv/Pas background. This result was instrumental for the subsequent experiment performed to analyze the phenotype of PI3kg KD mice. This allowed us to explore also the phenotype of PI3Kg KD mice, by using the appropriate strain-matched control of both WT and PI3Kg KO mice in the C57Bl/6J background.

8. Labels for antibodies used in immunohistochemical experiments in Fig 1A (and other similar figures) are not visible. Please improve.

We thank the Reviewer for noticing that. We have modified the figures.

2nd Editorial Decision 04 March 2015

Thank you for the submission of your revised manuscript to EMBO Molecular Medicine. I would like to particularly congratulate you on the provision of a very detailed M&M regarding animal use and statistical reporting!

We have now received the enclosed reports from the referees that were asked to re-assess it. As you will see the reviewers are now supportive and I am pleased to inform you that we will be able to accept your manuscript pending final editorial amendments:

Please submit your revised manuscript within two weeks.

I look forward to reading a new revised version of your manuscript as soon as possible.

***** Reviewer's comments *****

Referee #1 (Remarks):

The authors addressed my concerns satisfactorily.

Referee #2 (Remarks):

The authors have overall addressed my major concerns.
